# Polymer Blends Based on 1-Hexadecyl-3-methyl Imidazolium 1,3-Dimethyl 5-Sulfoisophthalate Ionic Liquid: Thermo-Mechanical, Surface Morphology and Antibacterial Properties

**DOI:** 10.3390/polym15040970

**Published:** 2023-02-16

**Authors:** Daniela C. Zampino, Filippo Samperi, Monique Mancuso, Tiziana Ferreri, Loredana Ferreri, Sandro Dattilo, Emanuele F. Mirabella, Domenico C. Carbone, Giuseppe Recca, Andrea A. Scamporrino, Elisabetta Novello, Concetto Puglisi

**Affiliations:** 1 Institute of Polymers, Composites and Biomaterials (IPCB)-CNR, Section of Catania, Via Paolo Gaifami, 18, 95126 Catania, Italy; 2 Institute for Biological Resources and Marine Biotechnology (IRBIM)-CNR, Section of Messina, Spianata San Raineri, 86, 98122 Messina, Italy; 3 Department of Integrative Marine Ecology, Stazione Zoologica “Anton Dohrn”, Sicily Marine Centre, Contrada Porticatello, 29, 98167 Messina, Italy; 4 Institute of Biomolecular Chemistry (ICB)-CNR, Section of Catania, Via Paolo Gaifami, 18, 95126 Catania, Italy

**Keywords:** antimicrobial polymer/ionic liquid blends, thermal properties, SEM, static contact angle, mechanical properties, *Staphylococcus epidermidis*, *Escherichia coli*

## Abstract

In this study, antibacterial polymer blends based on Polyvinyl Chloride (PVC) and Polystyrene-Ethylene-Butylene-Styrene (SEBS), loaded with the ionic liquid (IL) 1-hexadecyl-3-methyl imidazolium 1,3-dimethyl 5-sulfoisophthalate (HdmimDMSIP) at three different concentrations (1%, 5%, and 10%), were produced. The IL/blends were characterized by their thermo-mechanical properties, surface morphology, and wettability. IL release from the blends was also evaluated. The agar diffusion method was used to test the antibacterial activity of the blends against *Staphylococcus epidermidis* and *Escherichia coli*. Results from thermal analyses showed compatibility between the IL and the PVC matrix, while phase separation in the SEBS/IL blends was observed. These results were confirmed using PY-GC MS data. SEM analyses highlighted abundant IL deposition on PVC blend film surfaces containing the IL at 5–10% concentrations, whereas the SEBS blend film surfaces showed irregular structures similar to islands of different sizes. Data on water contact angle proved that the loading of the IL into both polymer matrices induced higher wettability of the blends’ surfaces, mostly in the SEBS films. The mechanical analyses evidenced a lowering of Young’s Modulus, Tensile Stress, and Strain at Break in the SEBS blends, according to IL concentration. The PVC/IL blends showed a similar trend, but with an increase in the Strain at Break as IL concentration in the blends increased. Both PVC/IL and SEBS/IL blends displayed the best performance against *Staphylococcus epidermidis*, being active at low concentration (1%), whereas the antimicrobial activity against *Escherichia coli* was lower than that of *S. epidermidis.* Release data highlighted an IL dose-dependent release. These results are promising for a versatile use of these antimicrobial polymers in a variety of fields.

## 1. Introduction

Microbial contamination affects various sectors, including medical, industrial, food, and domestic fields. Antibiotic resistance is one of the major threats in human society [1]. The emergence of antibiotic resistance is occurring worldwide, endangering the efficacy of antibiotics and becoming a great concern for public health, in particular for hospitals [2]. The increase in the prevalence of multidrug-resistant pathogens is occurring at a time when the discovery and development of new anti-infective agents is slowing down dramatically [2,3]. The World Health Organization of the United Nations (WHO-UN) has reported an increase in bacterial resistance in SSTIs, highlighting that the phenomenon regards Gram-negative pathogens such as *E. coli* [4]. In order to avoid the excessive use of antibiotics, research on alternative biocides, both natural [5,6,7] and synthetic with original structures [8,9,10,11,12,13,14], has recently been increasing. 

In recent decades, among biocide alternatives to antibiotics, ionic liquids (ILs) have been extensively studied. Many of these salts are liquid at room temperature and mainly consist of large nitrogen- or phosphorous-bearing cations, having one or more alkyl substituents, and inorganic or organic anions, such as halides, hexafluorophosphate, tetrafluoroborate, thiocyanate, dicyanammide, docusate, etc. [15]. The most important characteristic of ILs is their tunability, consisting of the possibility to design and synthetize many ILs with different physical, chemical, and biological properties simply by varying cations and anions [15].

ILs have been widely studied for different applications, thanks to their main characteristics such as negligible vapor pressure, thermal stability, low/no flammability, biocompatibility, antimicrobial activity, solvation capacity, high electrical conductivity, etc. [16].

Their application in “green” chemistry has been proposed to replace conventional organic solvents and volatile organic blends (VOCs) in chemical industry [15]. Some studies, indeed, have highlighted the need to substitute conventional organic solvents, often toxic, with ILs that have a lower toxicity [17].

ILs based on imidazolium salts have been used in a wide range of biological applications to produce surfactants, oxygen transport membranes, scaffolds for biomimetic applications, plasticizers, and antimicrobial and anti-inflammatory agents [9]. Antimicrobial ILs based on imidazolium and pyridinium rings have been developed and tested on different bacteria strains [9,15,18]. Moreover, it was found that ILs based on imidazolium exhibited strong antimicrobial activity, mainly due to the alkyl chain length in the cation. Actually, imidazolium salts comprising long alkyl chains (11–16 methylene groups) easily interact with the bacteria cell membrane, affecting its permeability and causing consequent cell death [8].

The aim of this study was the preparation of polyvinyl chloride (PVC) and polystyrene-ethylene-butylene-styrene (SEBS) blends, loaded with three different concentrations (1%, 5%, 10%) of the antibacterial IL 1-hexadecyl-3-methylimidazolium 1,3-dimethyl 5-sulfoisophthalate (HdmimDMSIP). PVC and SEBS were selected for their wide use in many applications across different industrial sectors. We used PVC plasticized with TOTM (referred to as PVC) to avoid health concerns due to the easy phthalate release into the environment and human fluids. Moreover, we chose the SEBS polymer because it is a phthalate-free and environmentally friendly thermoplastic elastomer that has been replacing PVC in multiple applications. Indeed, SEBS is easy to process, sterilizable, strong, and flexible and shows excellent heat and UV resistance. These characteristics make SEBS a valuable alternative to PVC in many industrial fields, such as medical, pharmaceutical, cosmetics and personal care, clothing, toys, sports adhesives and sealants, building materials, cables and wires, etc. [19]. 

Previous studies reported the loading of ILs into polymers as additives, processing aids, plasticizers, surfactants in the preparation of functional polymers, novel electrolytes in batteries, etc. [20]. They have been added into PMMA, PVC, PBT, PVA, PC, PET, and Pebax^®^ Rnew matrices as plasticizer [21,22,23], dispersant [24], and/or antimicrobial agents [11,12,25,26,27,28]. Nevertheless, to the author’s knowledge, the studies on antimicrobial SEBS blends do not include the loading of ILs into SEBS matrices as antimicrobial agents.

**Scheme 1:**
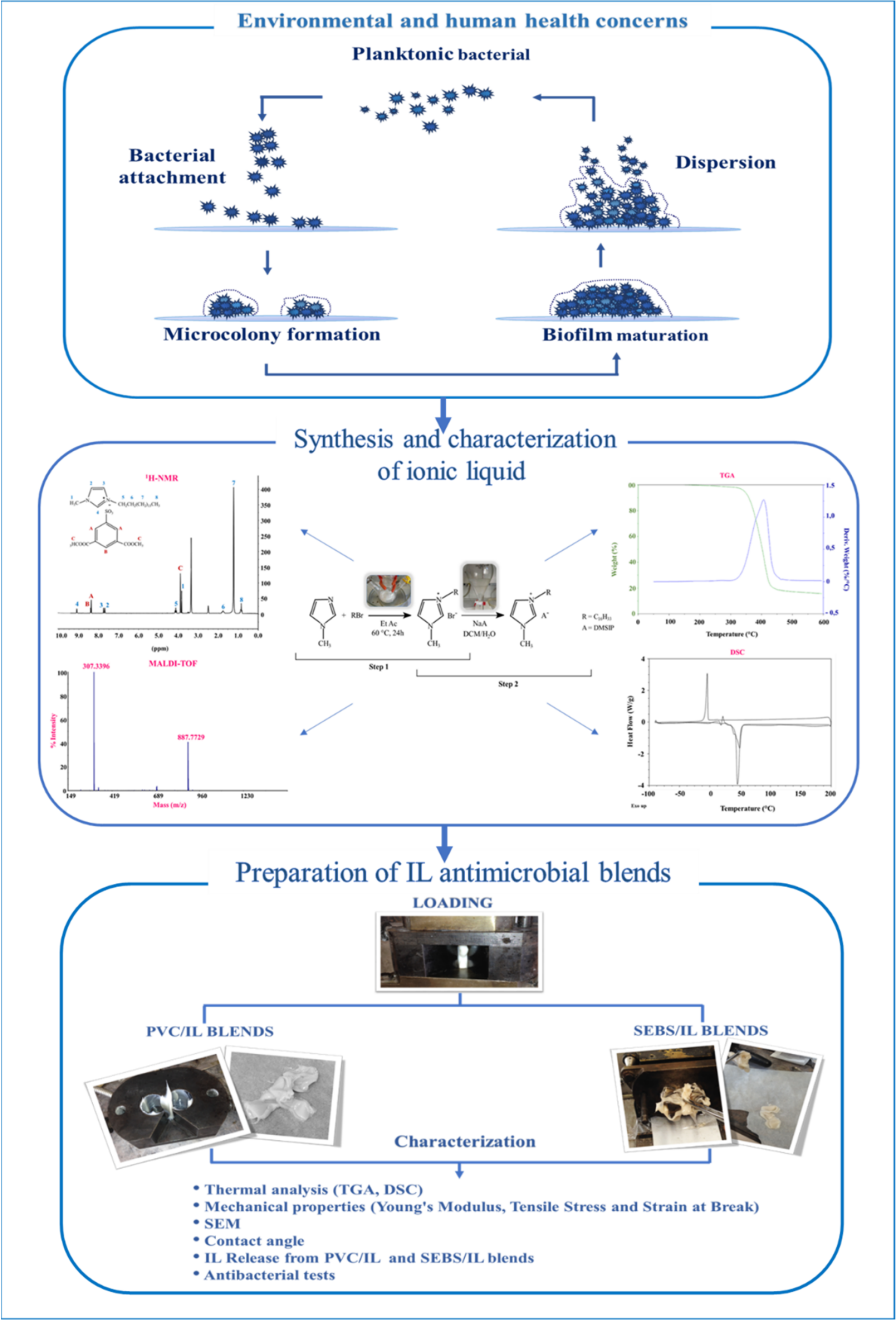
Focus of the study and research activities.

The main goal of the present study was the production of materials loaded with an antibacterial agent to be used in the manufacture of biomedical products. To this end, the IL HdMImDMSIP was synthetized and characterized. Both PVC/IL and SEBS/IL blends and their related films were obtained via melt mixing and successive compression molding. Thermal and mechanical properties together with antimicrobial activity, IL surface distribution, and wettability of the antimicrobial film blends were studied. The disk diffusion test was used to determine the antibacterial activity of the blend film samples against two bacterial strains, *Escherichia coli* and *Staphylococcus epidermidis*. They were chosen as representative of Gram-negative and Gram-positive human commensal bacteria that, under certain conditions, can transform from commensal to opportunistic pathogens, causing damage in catheterized, immunocompromised, surgically transplanted subjects. In Scheme 1, the focus of the study and the related investigations are detailed.

## 2. Materials and Methods

### 2.1. Materials

PVC plasticized with tris (2-ethylhexyl) trimellitate (TOTM), having a hardness (shore A) of 87.5, specific gravity of 1.242, and K-value of 65.0, and SEBS, with a hardness (shore A) of 80 and specific gravity of 0.89 were supplied by Consorzio Proplast (Alessandria, Italy). 1-methylimidazole (purity 99%, MW 82.10 g/mol), 1-bromohexadecane (purity 98%, MW 305.35 g/mol), 1,3-dimethyl 5-sulfoisophthalate sodium salt (purity 98%, MW 296.23 g/mol), dichloromethane (DCM) (purity 99.9%, MW 84.93 g/mol), ethyl acetate (purity 99.5%, MW 88.11 g/mol), tetrahydrofuran (THF) (purity 99.9%, MW 72.11 g/mol), dimethyl sulfoxide-d6 (DMSO-d6) (purity 99.8%, MW 84.17 g/mol), and trans-2-[3-(4-tert-butylphenyl)-2-methyl-2-propenylidene]malononitrile (DCTB) (purity 98%, MW 250.34 g/mol) were purchased by Sigma-Aldrich (Milan, Italy) and used as received. 

### 2.2. HdmimDMSIP Synthesis

**Scheme 2:**
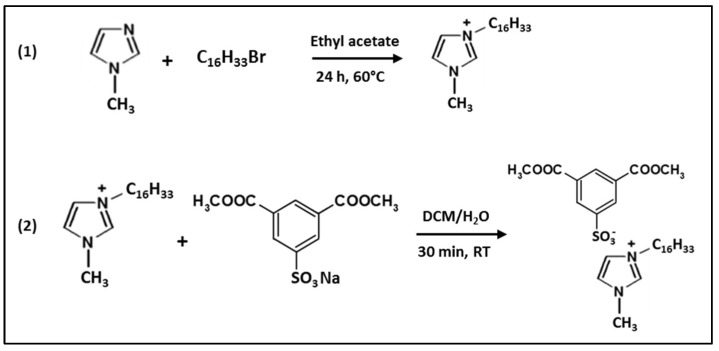
HdmimDMSIP synthesis. (1) First step; (2) second step.

The synthesis description of the ionic liquid 1-hexadecyl-3-methylimidazolium 1,3-dimethyl 5-sulfoisophthalate (HdmimDMSIP) was previously reported [11,12]. Scheme 2 displays the two steps of the synthesis.

During the first step, the IL 1-hexadecyl-3-methylimidazolium bromide (HdmimBr) was synthetized. 

In detail, 1-bromohexadecane (6.4 mL, 20 mmol) was made to react with an ethyl acetate solution (4 mL) of 1-methylimidazole (1.56 mL, 20 mmol) for 24 h at 65 °C under both stirring and a controlled atmosphere (nitrogen). The obtained compound was cooled down to room temperature, filtered, washed with ethyl acetate, and dried under vacuum at 40 °C for 24 h. The 1-hexadecyl-3-methylimidazolium bromide (HdmimBr) obtained was a solid white powder (Yield 95%; MW 387.44 g/mol).

^1^H NMR (400 MHz, DMSO-d6, δ ppm): 0.84 (t, 3H, CH3-C15), 1.23 (m, 26H, CH2), 1.77 (m, 2H, CH2-CH2-N), 3.85 (s, 3H, CH3-N), 4.15 (t, 2H, CH2-N), 7.72 (s, 1H, CH in imidazolium ring), 7.78 (s, 1H, CH in imidazolium ring), 9.15 (s, 1H, N-CH-N in imidazolium ring). 

The second step of the synthesis was a metathesis reaction between the obtained HdmimBr and the 1,3-dimethyl 5-sulfoisophthalate sodium salt. The reaction was carried out by placing a water solution (130 mL) of 1,3-dimethyl 5-sulfoisophthalate sodium salt (20.9 mmol, 6.03 g) together with a solution of HdmimBr (20 mmol, 7.77 g) dissolved in DMC (40 mL) in a separating funnel and energetically shaking at room temperature (25 °C). When no precipitate was observed in the resulting two-phase mixture (after ca. 30–45 min), the organic layer was recovered, dried over magnesium sulphate, and filtrated, and the residual solvent was taken out using a rotary evaporator. The obtained product was washed with ethyl acetate and dried at 40 °C in a vacuum stove for 24 h. A silver nitrate test was used to check that the bromide counter-ion exchange was completed. The obtained HdmimDMSIP was a white powder (Yield: 93.0%, MW: 580.78 g/mol).

^1^H NMR (400 MHz, DMSO-d6, δ ppm): Signals of imidazolium ring and alkyl chain: 0.84 (t, 3H, CH3-C15 chain), 1.22 (m, 26 H, CH2), 1.75 (m, 2H, CH2-CH2-N), 3.83 (s, 3H, CH3-N), 4.14 (t, 2H, CH2-N), 7.69 (s, 1H, CH in imidazolium ring), 7.75 (s, 1H, CH in imidazolium ring), 9.10 (s, 1H, N-CH-N in imidazolium ring). Signals of benzene ring: 3.90 (s, 6H, CH3-O), 8.37 (d, 2H, CH, ortho position with respect to SO3-substituents), 8.42 (d, 1H, CH, para position with respect to SO3-substituents).

### 2.3. Blends and Films Preparation

Before the films’ preparation, polymer pellets and IL powder were dried at 50 °C under vacuum for 24 h. PVC/HdmimDMSIP (PVC/IL) and SEBS/HdmimDMSIP (SEBS/IL) blends were obtained by mixing polymer pellets and the IL powder at different amounts (1, 5 and 10 wt %), in a Brabender mixer, according to the following operative mixing conditions: temperature 150 °C, speed 40 rpm, time 3 min and temperature 180 °C, speed 60 rpm, time 4 min were used for PVC/IL and SEBS/IL blends, respectively. Films of PVC/IL blends were obtained using compression molding at 150 °C (Time 10 + 2 min, Pressure 100 barr), whereas those of SEBS/IL blends were prepared at 180 °C (Time 10 + 2 min, Pressure 150 barr). The film samples (thickness 120–150 µm) were stored under vacuum to prevent moisture adsorption. 

### 2.4. Characterization

#### 2.4.1. Differential Scanning Calorimetry (DSC)

Calorimetric measurements were performed using a differential scanning calorimeter (DSC, TA Instruments Q100, Milano, Italy) equipped with a liquid sub-ambient accessory and calibrated with high purity standards (indium and cyclohexane). Nitrogen was used as purge gas. To evaluate all thermal transitions of neat polymers and their IL blends, heating and cooling cycles from −90 to 200 °C and vice-versa at a heating/cooling rate of 10 °C/min were executed. Firstly, a heating scan from 0 up to 200 °C was carried out to delete the previous thermal history. Samples weights were in the range of 4–6 mg.

#### 2.4.2. Thermogravimetric Analysis (TGA)

The thermogravimetric analyses were executed using a thermogravimetric apparatus (TGA, TA Instruments Q500) under a nitrogen atmosphere (flow rate 60 mL/min) at a 10 °C/min heating rate, from 40 °C to 600 °C. The weight loss percentage and its derivate (DTG) were recorded as a function of temperature. Sample weights were approximately 4–5 mg.

#### 2.4.3. Pyrolysis-Gas Chromatography/Mass Spectrometry (Py-GC/MS)

The Py-GC/MS analyses were performed using a Multi-Shot Pyrolyzer (EGA/PY-3030D, Frontier Labs, Saikon, Koriyama, Fukushima, Japan), joined to a GC system GC-2020 (Shimadzu Corporation, Kyoto, Japan) connected to a triple quadrupole mass spectrometry TQ8040 (Shimadzu Corporation) (electronic ionization 70 eV). An Ultra Alloy^®^ Metal Capillary Column (Frontier Labs, Saikon, Koriyama, Fukushima, Japan, stationary-phase 5% di-phenylmethylpolysiloxane, with an inner diameter of 250 µm, a film thickness of 0.25 µm, and a length of 30 m) was used. The interface of Py-GC was kept at 300 °C, whereas that of GC/MS was maintained at 250 °C. Samples were analyzed without pre-treatment, placing about 0.1 mg of them in the crucible (quartz capillary sample holder). The temperatures of 220, 250, 300 and 450 °C were used according to the thermal behavior of each sample determined using TGA analysis, in order to characterize the pyrolysis product formed at different temperatures. To obtain the GC separation, the following temperature program was used: isotherm at 50 °C for 1 min; heating from 50 to 100 °C at 30 °C/min; isotherm at 100 °C for 5 min; heating from 100 to 300 °C at 10 °C/min; and finally, isotherm at 300 °C for 10 min. The flow of the carrier gas (helium) was 1.78 mL/min, and the split ratio was 1/10 of the total amount of the carrier gas. Mass spectra were recorded under electron impact ionization energy at 70 eV, and the flow rate was kept constant. The MS detector was scanned from 30 to 500 m/z, at a scan rate of 2500 scans. Before each analysis, blanks were performed by placing the crucible empty in the furnace and applying the pyrolysis program adopted for sample analysis. Data analyses were performed with the LabSolution GC/MS Analysis software (Shimadzu Corporation, Kyoto, Japan).

#### 2.4.4. Mechanical Properties

The determination of the mechanical properties of the blends was performed using a tensile test machine (Zwick/Roell Z050) according to ASTM D882. Young’s modulus E, tensile strength (σ), and elongation at break percentage (ε) were evaluated. At least five replicates from each blend sample were tested. 

#### 2.4.5. Scanning Electron Microscopy (SEM)

The analysis of surface morphology of neat PVC and SEBS and their blends loaded with the three different concentrations of IL was performed using a Thermo Phenom Prox desktop SEM with an integrated energy-dispersive X-ray (EDS) detector. Before SEM analysis, samples were placed in carbon tapes, dried, and sputter-coated with gold. Images were acquired at 15 KV and a working distance (WD) of 6.3–6.9 mm.

#### 2.4.6. Contact Angle (CA)

Wettability of the blend films was determined at room temperature (25 °C) using a DATAPHYSICS OCA 15EC apparatus and water as liquid. The volume of the deionized water drop (2 µL) deposited on the sample surface was adjusted by the software of the optical tensiometer. After stabilization of the drop (~10 s), three measurements on different areas of the same sample were executed. The CA results are the average values of the three different measurements made on each sample (standard deviation ± 1–2.4°).

#### 2.4.7. IL Release from the Film Blends

The release kinetic of HdmimDMSIP from the films (1, 5, 10 wt %) was determined by immersing a rectangular sample (4 × 6 mm, 5 mg, 120–150 µm) in 3 mL of distilled water. The samples with and without the IL were kept at 37 °C; then, at specific interval times, the optical absorption at 210 nm was measured. By subtracting the absorption of the neat samples, the absorbances of the PVC/IL and SEBS/IL blend samples were converted to the amount of HdmimDMSIP released, based on a calibration curve (R^2^ = 0.999). The release experiments were performed in triplicate.

#### 2.4.8. Bacterial Strains and Growth Conditions

The *Escherichia coli* and *Staphylococcus epidermidis* strains were isolated from the human urinary tract at the Department of Biomedical and Dental Sciences and Morphofunctional Imaging, University of Messina, and used for the antibacterial assays.

Prior to the analysis, the strains were spread on Tryptic Soy Agar medium (TSA, Oxoid) and incubated at 37 °C for 24 h. Then the bacteria were collected, suspended in sterile saline solutions, and their concentrations adjusted to 10^6^ cell/mL using the McFarland tube.

#### 2.4.9. Antibacterial Screening 

The direct activity of the neat HdmimDMSIP IL was performed with the broth dilution method in order to determine its Minimal Inhibitory Concentration (MIC) vs both the *E. coli* and *S. epidermidis* strains. An initial IL solution was prepared at a concentration of 1% (*w*/*v*) by dissolving IL powder in sterile saline water at 80–100 °C under stirring for 5 min. Soon thereafter, serial dilutions from 1 mg/mL to 1 µg/mL were made.

The antibacterial activity of the blends was performed using the agar diffusion method [29]. Before microbiological analyses, samples of PVC/IL and SEBS/IL films, containing 1, 5, and 10% (*w/w*) of HdmimDMSIP, and samples of neat polymers (blanks) were sterilized with a UV lamp (wavelength 280–240 nm) through two steps of 3 min, according to the 2011 protocol of Zampino et al. [10]. 

Both bacterial strains were spread on TSA plates at a concentration of 10^6^ cell/mL. The film samples were placed on the TSA plate surfaces and incubated at 37 °C for 24 h. At least three replicates for each sample containing different concentrations of IL were performed. The antimicrobial activity of the blends was determined by measuring the width (mm) of the inhibition halos of bacteria growth. 

## 3. Results

### 3.1. Synthesis and Characterization of HdmimBr and HdmimDMSIP ILs

The HdmimDMSIP IL was synthetized using a two-step method (Scheme 1). An extensive description of its synthesis and characterization by ^1^H Nuclear Magnetic Resonance spectroscopy (^1^H NMR) and Matrix Assisted Laser Desorption Time of Flight Mass Spectrometry (MALDI TOF MS) analyses was previously reported [11,12]. Both ^1^H NMR and MALDI TOF MS analyses confirmed that imidazole ring functionalization (first step) and ion exchange (second step) took place. 

In Table S1, the chemical structures and the measured and calculated exact masses of ILs synthetized are reported. The signals at m/z 307.41 and 307.34 are relative to the cationic species, whereas those at m/z 694.99 and 887.77 correspond to adducts consisting of IL and cation relative to HdmimBr and HdmimDMSIP ILs, respectively.

### 3.2. Blend Film Preparation

Before preparation of the membranes, polymer pellets and IL powder were subjected to a drying procedure (40 °C for 24 h in a vacuum oven). The antimicrobial blends and films were obtained by melt mixing and compression molding, respectively. Film thickness was ~120–150 µm. After different attempts, the IL HdmimBr was not used as antimicrobial agent due to its lower thermal stability (Figure S1) than that of HdmimDMSIP (Figure S2), determined by TGA. Its addition to both polymer matrices caused initial thermal degradation and yellowing-browning of the samples under the operating conditions adopted.

### 3.3. Thermal Analysis

Easily loading biocides into the polymer matrices is very important for the industrial production of antimicrobial polymers, avoiding difficult synthesis processes. To this end, the chemical and physical properties of the antibacterial agents to be used are essential because, in addition to their peculiar bacteriostatic/bactericidal features, they must possess characteristics compatible with the molding and processing technologies adopted during the industrial manufacture of end products. Moreover, during polymer processing, degradation routes may occur, affecting the properties and the performance of the formed materials. Consequently, the investigation of the thermal stability and properties of biocides becomes of fundamental importance in planning and obtaining industrial products suitable for different uses. In this study, the analysis of thermal properties and the stability of IL and related polymer compounds was carried out using DSC and TGA measurements.

#### 3.3.1. DSC

The DSC analysis was performed using a temperature range from −90 °C to 200 °C and heating/cooling rates of 10 °C/min. The DSC analysis of HdmimBr revealed a temperature of fusion (T_f_) of 68.0 °C and a crystallization temperature (T_c_) of 37.0 °C, whereas the IL HdmimDMSIP showed T_f_ and T_c_ values of 49.0 °C and −4.1 °C, respectively. During the second heating cycle, both ILs showed an exothermic peak due to cold crystallization (T_cc_), and a polymorphic behavior, similar to that of polymer materials, as previously reported [11,12,30,31]. Glass-transition temperature (T_g_) was not observed for either IL (Figures S1 and S2).

As phthalate exposure determines environmental and human health concerns, for all experiments we used a formulation of PVC plasticized with TOTM.

**Figure 1:**
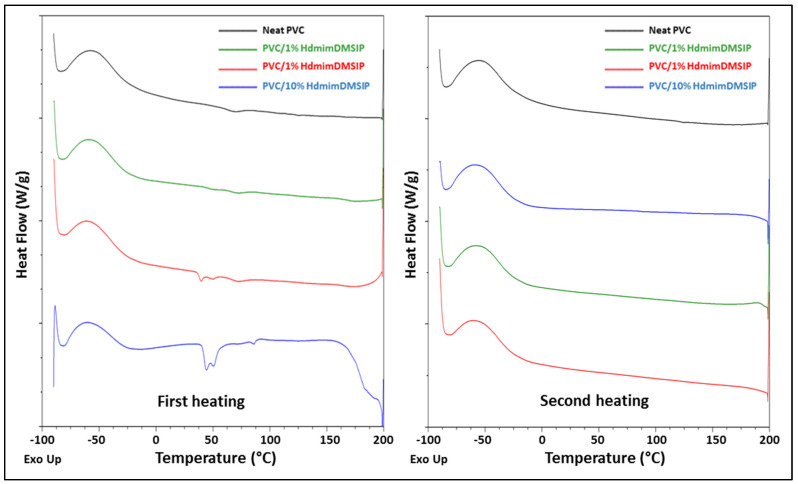
DSC curves of neat PVC and PVC/HdmimDMSIP blends. Curves are displaced for clarity.

DSC curves of neat PVC and PVC/IL blends are reported in Figure 1. Neat PVC showed a glass-transition temperature (T_g_) of 63.0 °C during the first heating cycle, not detected during the second one. The T_g_ is observed in the PVC/IL blends containing 1–5% of HdmimDMSIP, whereas in PVC/10% IL it is not well defined. PVC blends loaded with 5–10% IL showed polymorphic melting peaks similar to those of pure IL, suggesting that these concentrations are excessive for good compatibility with the polymer matrix. Furthermore, as observed for neat PVC, blends curves do not show T_g_ and T_m_ transitions during the second heating scan, indicating that the primary plasticizer effect of TOTM overshadows the IL contribution [12].

**Figure 2:**
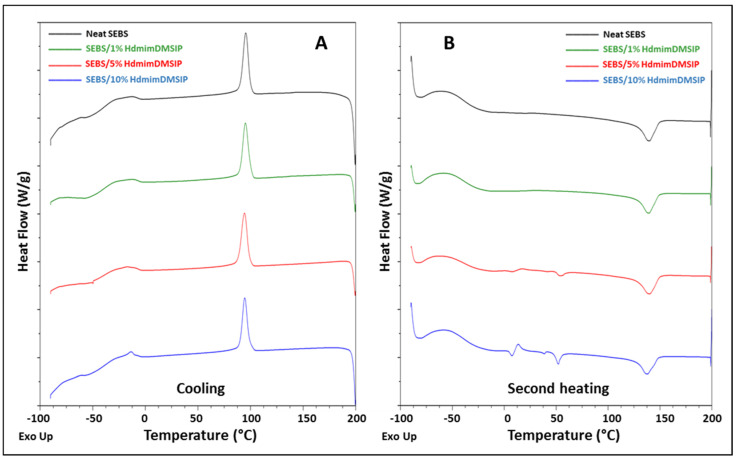
DSC curves of neat SEBS and SEBS/HdmimDMSIP blends during (**A**) the cooling and (**B**) the second heating runs. Curves are shifted for clarity.

During the second heating scan, DSC curves of neat SEBS and SEBS/IL blends (Figure 2) evidenced melting peaks of Styrene (S) blocks and T_g_ values of Ethylene Butylene (EB) blocks ranging from 140 °C to 138 °C and from −41 °C to −38 °C for neat SEBS and SEBS/10% IL, respectively. As observed for PVC/IL blends, additional endothermic transitions at 44 °C and 52 °C in the blends containing the IL at 5% and 10% concentrations, respectively, were detected.

The cooling scan showed the crystallization peaks of the Styrene blocks from 95.5 °C to 94.6 °C and the small and wide exothermic peaks due to crystallization transitions of the Ethylene Butylene blocks from −31.4 °C to −29.9 °C. The slight variations of glass transition, melting, and crystallization peaks suggest that the loading of HdmimDMSIP does not affect the thermal properties of SEBS, suggesting a phase separation of the IL in the block copolymer matrix [32].

#### 3.3.2. TGA

The short-term thermogravimetric analyses were carried out via heating/cooling runs of 10 °C/min from 40 to 600 °C [12]. A platinum pan and nitrogen gas were used to avoid degradation differences due to pan and gas types [30,33,34,35]. Moreover, we considered T_onset_ the temperature at 5% weight loss, as previously reported [34]. 

The ILs’ TGA data show that HdmimDMSIP is stable up to 300 °C, whereas HDMImBr is stable below 200 °C. Degradation of both ILs occurs in a single step, recording the maximum degradation temperature at 290 °C for the HDMImBr and at 407 °C for the HdmimDMSIP (Figures S3 and S4). Moreover, a higher residue (13.1) was registered from the degradation of HdmimDMSIP with respect to that of HdmimBr (0.1). HdmimBr showed its onset of degradation at 161 °C, probably due to the water/solvent retained even after a rigorous drying in a vacuum oven for 48 h. 

The hygroscopic behavior of HdmimBr affected its thermal stability; indeed, preliminary experiments performed adding this IL to PVC matrix yielded yellowish blends showing initial degradation. A similar behavior was observed for SEBS blends loaded with HdmimBr. Consequently, we chose to prepare both antimicrobial polymer blends adding only the HdmimDMSIP IL.

**Figure 3:**
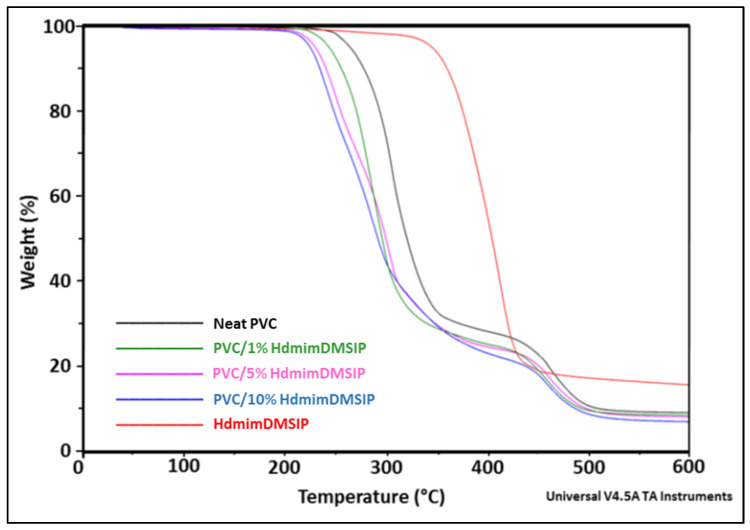
TGA curves of the IL HdmimDMSIP, neat PVC, and PVC/IL blends.

**Figure 4:**
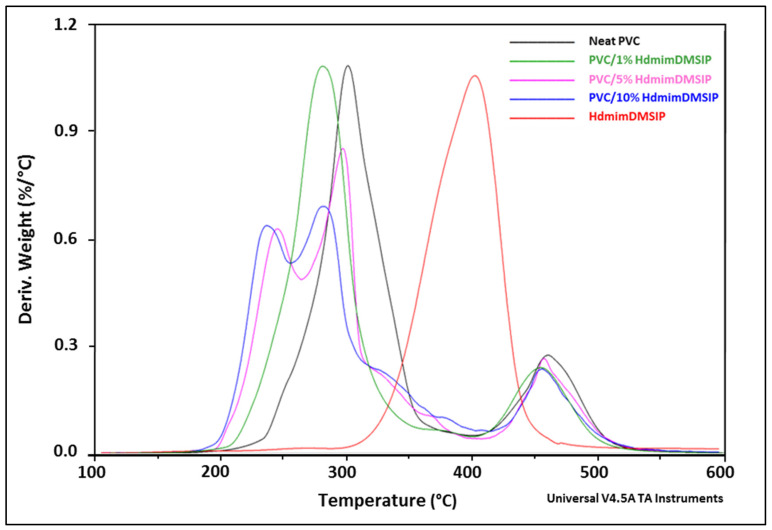
DTG curves of the IL HdmimDMSIP, neat PVC, and PVC/IL blends.

Figure 3 and Figure 4 report the TGA and DTG curves of HdmimDMSIP, neat PVC, and PVC/IL blends.

Neat PVC shows two thermal degradation steps. The maximum degradation temperature of the first step (Td_1_), at about 305 °C, is due to hydrochloric acid loss (~60% *w/w*), whereas the maximum degradation temperature of the second step (Td_2_), registered at ca. 457 °C, is due to degradation of the newly formed polyene chains [36]. At temperatures below 200 °C, no significant differences between neat PVC and PVC/IL blends were found, whereas at temperatures higher than 200 °C, all the PVC/IL samples showed a decrease of onset temperature with an increasing percentage of IL in the polymer matrix. In particular, the onset temperatures decreased 24, 39, and 49 °C with the addition of 1, 5, and 10% of HdmimDMSIP, respectively. Despite the significant decrease compared to neat PVC, the T_onset_ of the PVC/10% IL blend is above 200 °C (at 215.5 °C), thus it does not affect the thermal stability of PVC for many of its applications. The same decreasing trend was registered for the temperatures of the first step of degradation (Td_1_). The second step of thermal degradation (Td_2_) displayed only slight variations (up to 11 °C) between neat PVC and PVC/IL blends (Figure 3).

During the first step of degradation, we found a decrease of Td_1_ up to 78 °C with an increasing percentage of HdmimDMSIP. The lower thermal stability of PVC/IL blends is due to the IL taking part in the catalytic degradation of PVC, anticipating dehydrochlorination of chlorine atoms from PVC chains. In particular, the loading of IL at 5–10% concentrations induced a split of Td_1_ into two peaks (Figure 4), the first of which was due to the anticipated dehydrochlorination and the second one was due to the initial degradation of the IL. It was previously reported [25] that the difference in the thermal stability of PVC/IL blends could also be due to the different ILs solvating power, with the consequence that solvated parts of PVC undergo decomposition at lower temperatures. The introduction of ILs into other polymer materials induced a similar lowering of thermal decomposition. Indeed, the loading of ILs into the PC matrix decreased the decomposition temperature, inducing a two-step degradation process for PC/ILs compounds with respect to the single step displayed by pure PC [27]. 

The residue of PVC/IL blends, observed at 600 °C, decreased from 8.8% of neat PVC to 7.2%, proportionally to the amounts (1–5%, *w/w*) of IL loaded into the blends. The slightly higher residue (10%) of PVC/10% IL blend is probably due to the involvement of IL in the crosslinked complexes with the polyenic chains, which did not degrade up to 600 °C.

**Figure 5:**
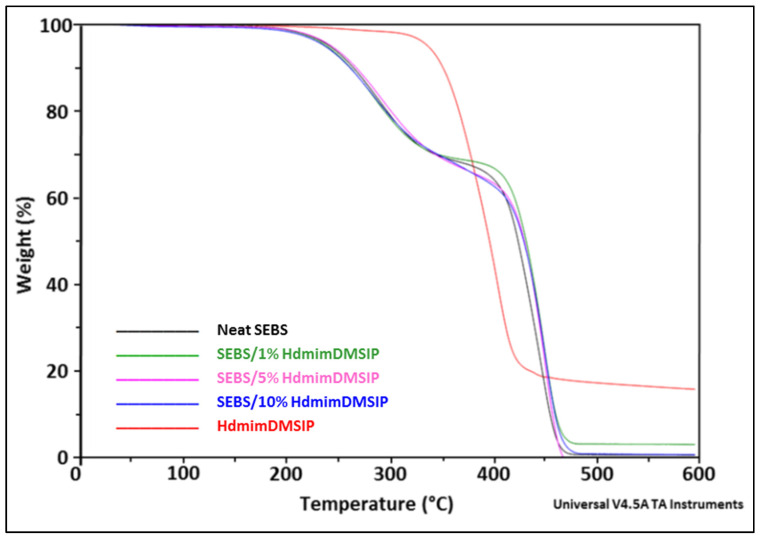
TGA curves of the IL HdmimDMSIP, neat SEBS, and SEBS/IL blends.

**Figure 6:**
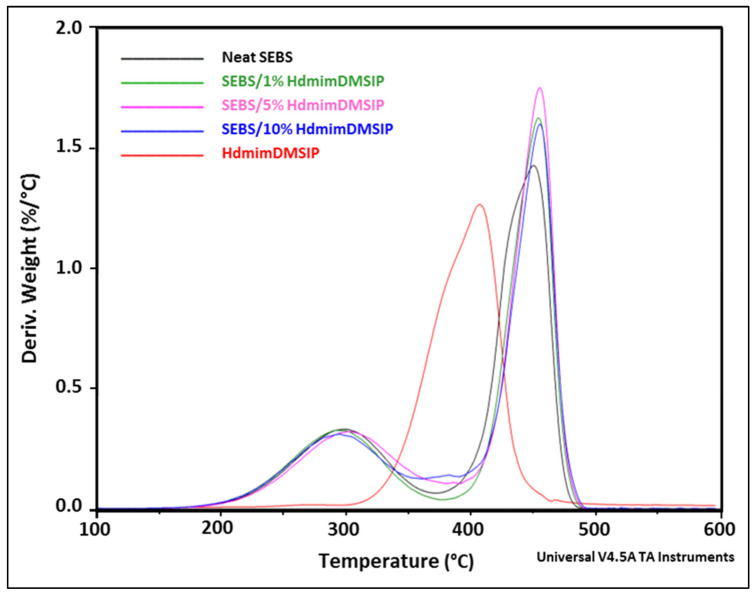
DTG curves of the IL HdmimDMSIP, neat SEBS, and SEBS/IL blends.

Neat SEBS also showed two steps of thermal decomposition (Figure 5 and Figure 6) at 301 °C (Td_1_) and at 443 °C (Td_2_), concerning the EB soft and the PS hard phases, respectively. No significant differences between neat SEBS and SEBS/IL blends were observed below 200 °C, whereas at temperatures higher than 200 °C, all the blends showed a slight decrease in the onset temperature (up to 8 °C). Considering the Td_1_, the addition of IL to the polymer matrix led to a 15 °C decrease in degradation temperature according to the increase in IL concentration, differently from the significant decrease in Td_1_ showed by the PVC/IL blends. No differences in the Td_2_ values were registered. These results are in agreement with literature data [11,12,26], showing a slight decrease up to a maximum of 8 °C with liquid ionic content. The temperatures of both maximum degradation rates (Td_1_ and Td_2_) were not affected by the addition of the IL into SEBS matrix, displaying values, for all the blends, higher than those of the polymer processing and the end-products’ usage. Similarly, the introduction of copper microparticles into SEBS/PP compounds did not induce significant changes in the thermal properties of the compounds [32].

At 600 °C, the pure SEBS is almost completely degraded, showing a residue of 0.45% which rises to 1% in the SEBS/IL blends, as the concentration of IL increases.

**Figure 7:**
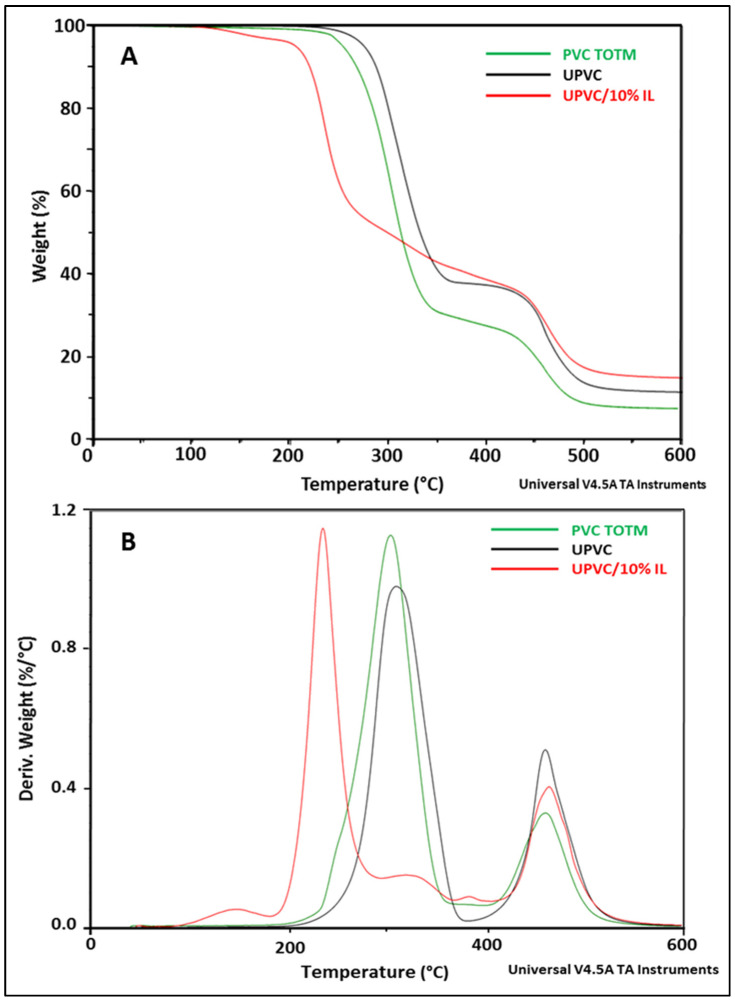
Overlay of TGA (**A**) and DTG (**B**) curves of unplasticized PVC (UPVC), PVC plasticized with TOTM, and UPVC blend containing 10% HdmimDMSIP.

To determine the effective influence of the IL and/or the TOTM on the thermal degradation of PVC, we also analyzed neat unplasticized PVC (UPVC) and an UPVC blend loaded with 10% of IL (Figure 7) using TGA. Results from comparation of plasticized PVC with TOTM, UPVC and UPVC/10% IL blend demonstrated a higher influence of IL than TOTM in taking part in the first degradation step of PVC, favoring anticipation of dehydrochlorination mechanisms. 

### 3.4. Py-GC MS

To gain further knowledge about the degradation processes occurring during desorption of IL and its influence on the degradation process of its related polymer blends, we analyzed the degradation routes of all samples using Py-GC MS.

ILs mainly degrade via dealkylation of the imidazolium ring, following different routes. During Py-GC MS of ILs constituted by halides anions [37], the nucleophilic attacks of halide ions on the alkyl groups and the following C–N bond cleavage mainly determine the formation of imidazolium halides, haloalkanes, and 1-alkylimidazoles corresponding to the alkyl substituents and alkenes in minor amounts. The ILs with BF_4_, PF_6_ and CF_3_SO_3_ anions principally produce analogous alkenes and 1-alkylimidazoles rather than haloalkanes. The authors [37] reported that in the samples containing PF_6_, minor amounts of phosphorous-containing products are formed, whereas no boron-containing products are produced from the samples with BF_4_ anion. Moreover, in the samples with longer alkyl groups, pyrolyzate products derived from cleavages of the C–C bond in the alkyl groups are formed. Imidazole rings do not decompose up to about 550 °C. 

In-depth information on the composition and distribution characteristics of the pyrolysis products of IL, neat polymers, and both PVC/IL and SEBS/IL blends were achieved using Py-GC/MS analyses. To the best of the authors’ knowledge, there are no pyrolysis studies on the IL composed by the Hdmim cation and the anion DMSIP. 

**Figure 8:**
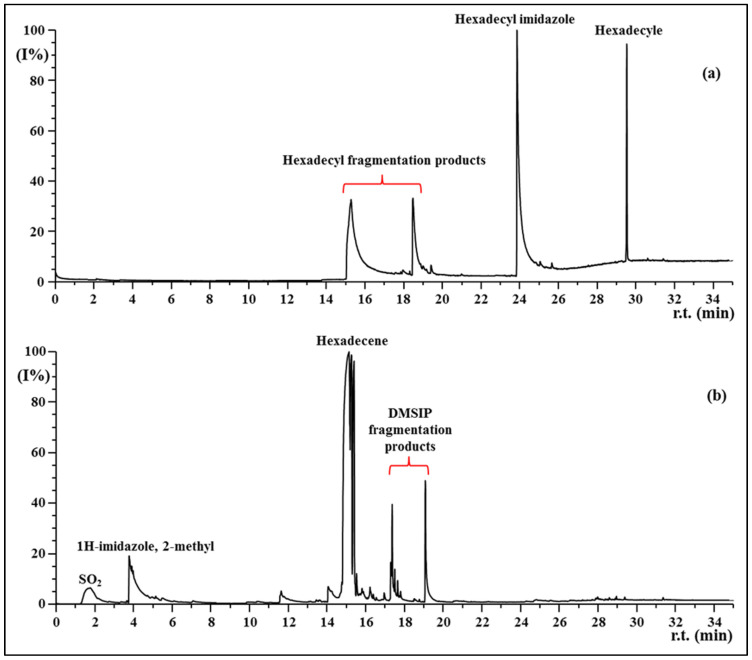
Py-GC/MS pyrograms of the thermal products of the IL HdmimDMSIP recorded at (**a**) 300 °C and (**b**) 450 °C.

In Figure 8, the pyrograms of the pyrolysis products of the IL HdmimDMSIP recorded both at 300 °C (Figure 8a) and at 450 °C (Figure 8b) are shown. The formation of methyl-imidazole and hexadecyl imidazole moieties was found at 300 °C. Pyrolysis products due to the C-C bond scissions in the hexadecyl group were also observed. These results suggest that the fragmentation of the IL occurs via dealkylation of the imidazolium ring, in agreement with literature data [34,37,38]. Moreover, the pyrogram at 450 °C (Figure 8b), in addition to the fragmentation products of the imidazolium cation, shows those due to 1,3-dimethyl 5-sulfoisophathalate (DMSIP) anion (range peaks at r.t. 17–20 min). 

**Figure 9:**
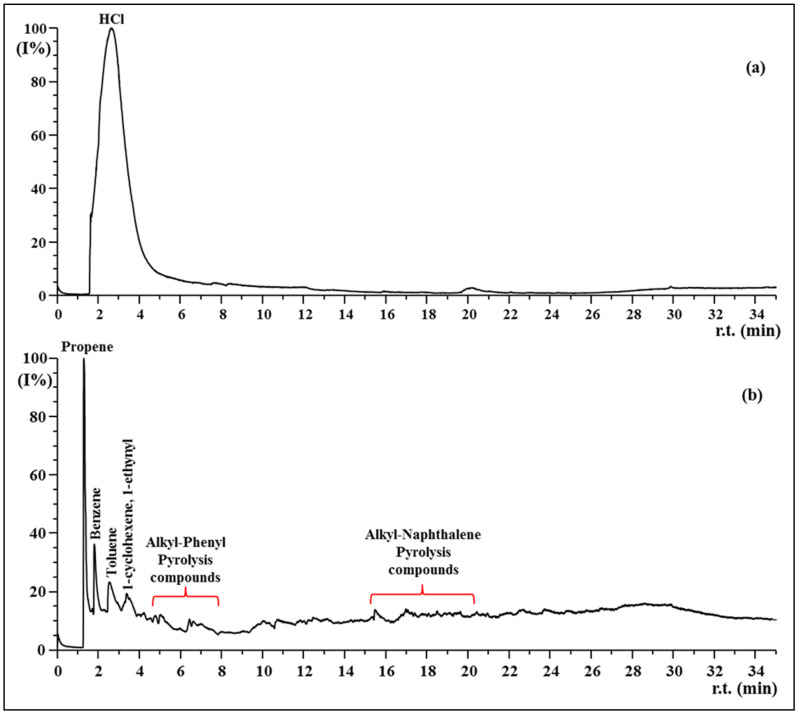
Py-GC/MS pyrograms of the thermal products of the unplasticized PVC (UPVC) recorded at (**a**) 300 °C and (**b**) 450 °C.

The pyrogram of the UPVC (Figure 9) recorded at 300 °C (Figure 9a) shows the predominant formation of HCl, confirming the dehydrochlorination process with the production of HCl and polyene chains that takes place during the first thermal degradation step, as observed by TGA (Figure 3 and Figure 4). In the second pyrolysis step with a maximum degradation temperature at about 450 °C, the fragmentation of the generated polyene chains leads to the formation of unsaturated hydrocarbons and aromatic compounds such as propine, 1-ethynil 1-cyclohexene, benzene, toluene, naphthalene, and alkyl naphathalenes (Figure 9b). 

**Figure 10:**
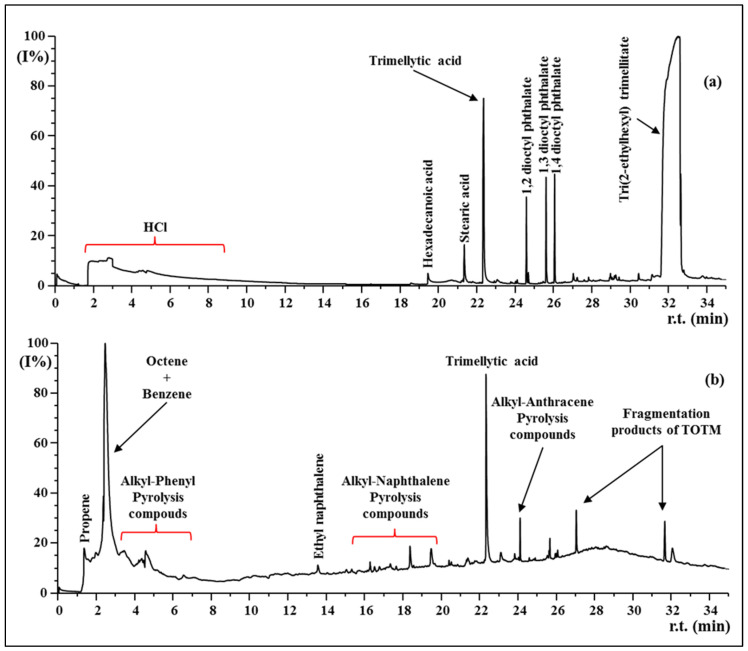
Py-GC/MS pyrograms of the thermal products of the plasticized PVC recorded at (**a**) 300 °C and (**b**) 450 °C.

Likewise, the thermal degradation of PVC plasticized with Trioctyl trimellitate (TOTM) (Figure 10) occurs in two stages with maximum rates of weight loss (Td_1_ and Td_2_) at about 300 °C and 450 °C (Figure 3 and Figure 4). However, the comparison between plasticized and non-plasticized PVC (Figure 9 and Figure 10) highlights peculiar differences. Indeed, the pyrogram of the plasticized PVC recorded at 300 °C (Figure 10a), in addition to the predominant formation of HCl, shows an intense peak due to undegraded TOTM and other peaks due to TOTM pyrolysis products, such as 1,2-di(2-ethylhexyl) phthalate (DEHP)), 1,4-di(2-ethylhexyl) phthalate (Dioctyl terephthalate), 1,3-di(2-ethylhexyl) phthalate (DOIP), and trimellitic acid (1,2,4 Benzene tricarboxylic acid). Our findings agree with literature data [39]. Indeed, the authors studying the thermo-oxidative stability of the TOTM plasticizer using Py-GCMS at 700 °C found the formation of DEHP and DOIP fragmentation products. 

We suppose that a part of the HCl formed by dehydrochlorination of the PVC chains could react with the TOTM, allowing the formation of its thermal degradation products (trimellitic acid, DEHP, DOIP and dioctyl terephthalate). Figure 10a also shows the presence of stearic acid. This is probably due to the common use of Ca, Zn, Mg, and Pb stearic acid salts either as lubricants or stabilizers of PVC. The pyrogram of the PVC recorded at 450 °C (Figure 10b) shows fragmentation products derived from the pyrolysis of the polyene chains formed by dehydrochlorination of the PVC and also from pyrolysis of the TOTM plasticizer.

**Figure 11:**
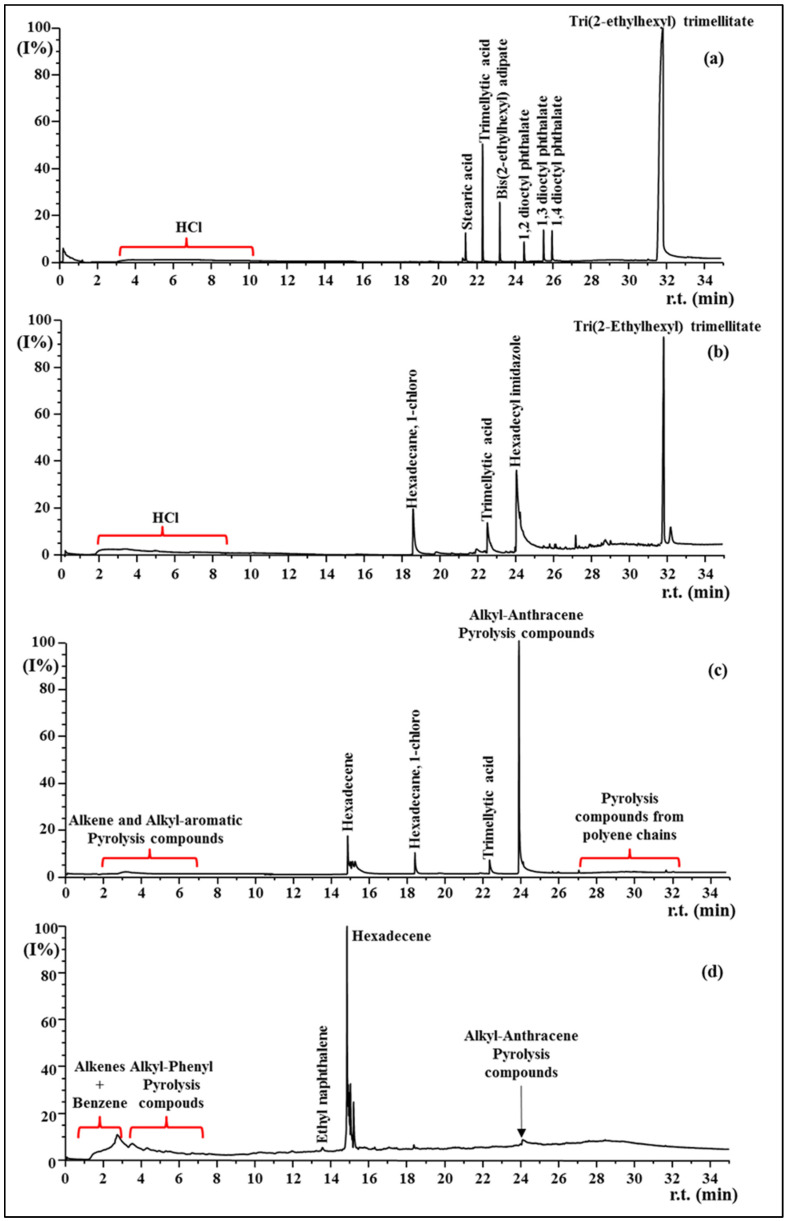
Py-GCMS pyrograms of the thermal products of the PVC/10% HdmimDMSIP blend at (**a**) 220 °C, (**b**) 300 °C, (**c**) 350 °C, and (**d**) 450 °C.

The Py-GC/MS pyrograms of the PVC/10% IL blend recorded at different pyrolysis temperatures are depicted in Figure 11. According to TGA data, the Py-GCMS analysis of the PVC/IL blends containing 1%, 5%, and 10% *w/w* of IL confirmed that Td_1_ decreases as the amount of IL increases (Figure 3 and Figure 4). In fact, the first thermal degradation products were observed at about 270 °C in the case of the PVC/1% IL blend (Figure S5), and already at about 200 °C for the PVC/10% IL blend (Figure 11). This confirms that the IL loading into the PVC matrix influences the dehydrochlorination process occurring during the first thermal degradation step of the polymer. The HCl formed at a lower temperature than that of neat PVC can react with the thermal degradation products of the IL, as suggested by the identification of hexadecane 1 chloro compounds at 300 °C. The pyrogram of PVC/10% IL blend recorded at 220 °C (Figure 11a) shows the formation of HCl and pyrolysis products of the TOTM. This result indicates that the anticipation of the dehydrochlorination process caused by the IL also determines the lowering of the thermal stability of the TOTM plasticizer, inducing its reaction with the formed HCl already at a relatively low degradation temperature (close to 200 °C). 

Very similar pyrograms were obtained analyzing the PVC/5% IL blend (Figure S6). 

**Figure 12:**
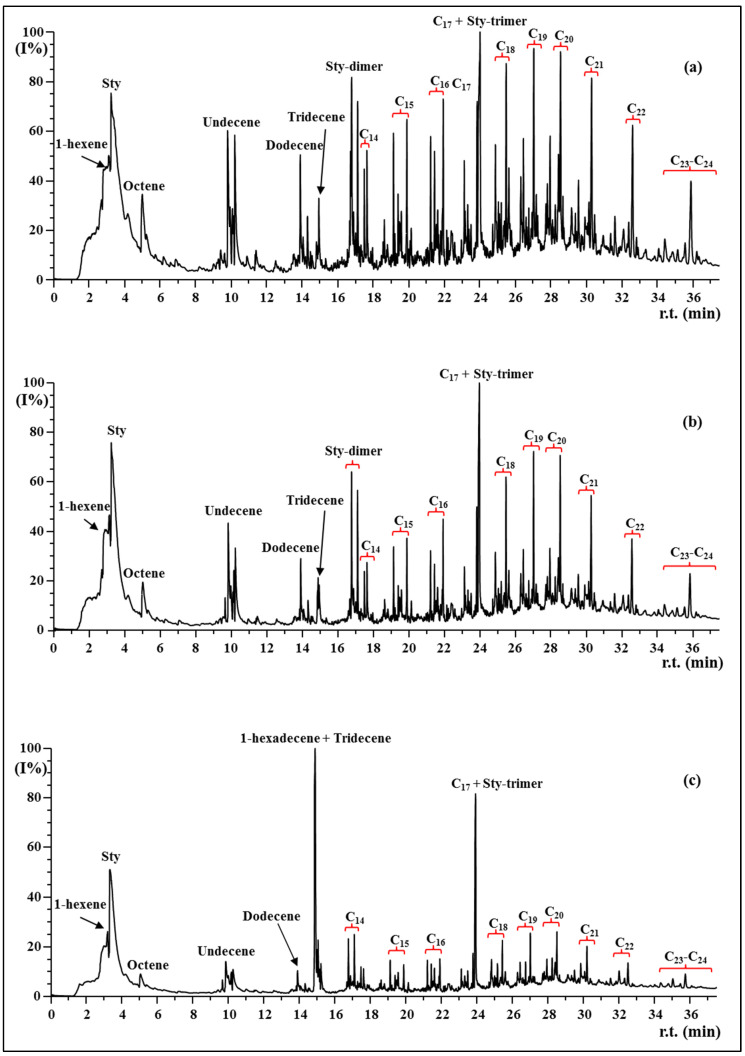
Py-GCMS pyrograms of the pyrolysis products of neat SEBS (**a**), SEBS/1% HdmimDMSIP, (**b**) and SEBS/10% HdmimDMSIP (**c**) at 450 °C.

As observed in Figure 5 and Figure 6, the addition of the IL did not promote significant changes in the thermal degradation behavior of the SEBS. This was also confirmed by Py-GCMS of neat SEBS and its blends (Figure 12, Figures S7 and S8). Indeed, broadly similar pyrograms were recorded from both neat SEBS and SEBS/IL blends pyrolyzed at 300 °C (Figure 12 and Figure S6). The GS-MS spectra suggest that, at this temperature, the thermal degradation of the EB copolymer chains of the neat SEBS occurs. The composition of SEBS block copolymer depends on the nature of the olefin starting material, their composition, and the manufacturing process [40]. The elastomer block consists of a copolymer of ethylene and butylene, which derives from a butadiene precursor via hydrogenation. 

The PY-GCMS pyrogram of neat SEBS at 450 °C (Figure 12a) shows that styrene (peak in the range 2–4 r.t. min) is formed owing to the thermal degradation mechanisms involving the polystyrene blocks, which also lead to the formation of either styrene dimer (peak at r.t. 16.7–17.1 min) or styrene trimer (peak at r.t. 23.9–24.1 min). The formation of styrene dimer and trimer was already observed in the thermal degradation of polystyrene-based polymers [41,42,43,44]. The other peaks present in the pyrogram of SEBS pyrolyzed at 450 °C were assigned to the alkenes and alkyl-alkenes C14–C22 generated from pyrolysis of the EB (ethylene-co-but-1-ene) blocks of the SEBS copolymer. Very little differences between the pyrograms of SEBS and SEBS/IL blends pyrolyzed at 450 °C were observed. A relative increase of the peak intensity at r.t. of 14.92 min and 23.92 min was observed as the amount of IL increased in the blends. The peak at 14.92 min was assigned to the hexadecene formed by thermal degradation of IL, whereas the peak at 23.92 min was assigned to the styrene trimer. The other pyrolysis compounds from IL were not distinguished because they were overshadowed by those generated from SEBS pyrolysis. Py-GCMS data suggest that SEBS and HdmimDMSIP degraded independently in their blends during heating. Although thermo-oxidative degradation of SEBS has been studied by thermal analysis, UV, luminescence, and FTIR spectroscopy [45], and the mechanisms occurring in each oxidation phase have been proposed, to the best of our knowledge, this is the first study on the thermal degradation of SEBS under inert conditions using the Py-GCMS technique. 

### 3.5. Mechanical Properties

The study of the mechanical properties of polymers is of intense interest due to the complex structure of polymeric materials and their relationships with the environment during their related product preparation, storage, distribution, and use. The mechanical characteristics depend on various factors, among which are chemical-physical properties (chemical nature, composition, crystallinity, molecular weight, branching, etc.) and environmental features. The processing methods (temperature, pressure, time, working atmosphere, etc.) can also influence the mechanical behavior of polymeric materials [46]. 

In the present study, we analyzed the stress–strain behavior of the polymers and their blend films in order to verify whether the addition of the IL into the PVC and SEBS matrices affect their mechanical performance. 

The influence of the HdmimDMSIP on the mechanical properties of PVC/IL and SEBS/IL melt mixed blends was studied according to the microtensile ASTM D882 using a tensile test machine (Zwick/Roell Z050). Films of both PVC/IL and SEBS/IL blends were prepared using compression molding, and the Young’s modulus (E), tensile strength (σ), and elongation at break percentage (ε) were measured. All data are summarized in Table 1. Figure S9 displays the changes of Young’s modulus, tensile strength and elongation at break percentages in the PVC/IL blends (Figure S9a–c) as IL% increases. It shows that increasing IL% determines a decrease in both values of E and σ and an increase in the ε, suggesting a plasticizing effect, according to the DSC data discussed above. Our results are in agreement with literature data [12,23]. In a previous paper on PVC blends loaded with the same IL HdmimDMSIP but prepared using *solvent casting* [12], a similar behavior of mechanical properties was found, indicating that the different preparation method adopted did not influence the mechanical behavior of the blends. Hou and Wang [23] reported that PVC resin plasticized with ILs had elongation at break enhanced, and tensile strength and elastic modulus decreased. They hypothesized that the increase of flexible side-chain length induces more soft characteristics, and consequently a decrease of elastic modulus. Moreover, they suggested that ILs exerted a lubrication action that increases material plasticity, weakening interface energy between PVC paste resin and ILs.

In the case of SEBS/IL (1%, 5%, 10%) blends, a slight decay of all mechanical properties (E, σ and ε) analyzed is observed as the amount of the IL increases (Figure S9d–f). This behavior can be caused by a non-homogeneity in the materials due to the incompatibility between SEBS and IL, as confirmed by DSC data. Sierra et al. [47] have observed that thermal and mechanical properties of SEBS copolymers depend on both styrene and ethyl branch levels. 

### 3.6. Scanning Electron Microscopy SEM

The SEM technique makes it possible to observe dynamic surface phenomena on the nanoscale and explores intricate patterns in nanostructured surfaces of polymer samples. This tool is widely used to investigate the surface structure, particle size and shape, filler orientation and dispersion, and fracture and failure mechanics in polymer matrices. In the present study, the SEM analysis of PVC and SEBS and of their blends made it possible to obtain information about the distribution of the IL in the polymer surfaces. This information was very important for better understanding the CA and the IL release results.

**Figure 13:**
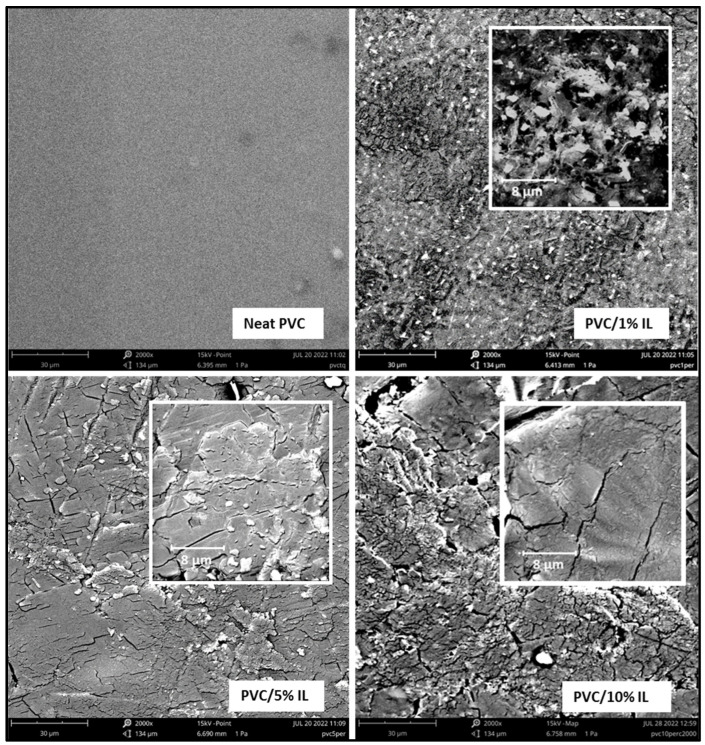
SEM images of neat PVC and PVC/HdmimDMSIP blends. Inset magnification 10,000×.

**Figure 14:**
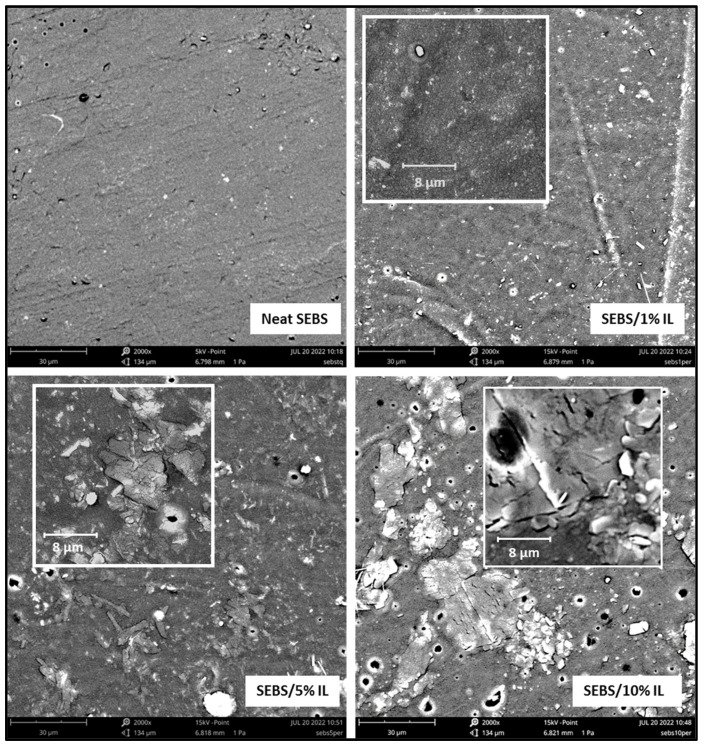
SEM images of neat SEBS and SEBS/HdmimDMSIP blends. Inset magnification 10,000×.

The surface morphologies of the film samples, detected by SEM, are shown in Figure 13 and Figure 14. As it can be seen from the SEM images, the IL introduction into both PVC and SEBS matrices plays an important role on the surface morphology of their related blend films. 

While neat PVC did not display any peculiar features, the loading of IL into PVC determines film surface modification depending on IL concentration (Figure 13). Indeed, the PVC/1% IL blend shows only a slight distribution of IL on the film surface, indicating that this concentration is suitable for a homogeneous distribution of the additive into the PVC matrix. PVC blends loaded with 5–10% of IL display relevant morphological modifications determined by the massive distribution of IL on their film surfaces. Irregular structures distributed broadly on the surface, showing roughness in more or less compact areas, are clearly visible. In the PVC/10% IL film blend, the surface segregation of the IL is more evident, exhibiting areas in which it is distributed in a wide and compact way, with the consequent tendency to fracture in thick blocks. This behavior is in agreement with a previous study on PVC loaded with HdmimDMSIP using *solvent casting* [12]. Nevertheless, the PVC/5–10% HdmimDMSIP blend films prepared using *solvent casting* showed an oriented and compact morphology, less evident in their related melt-mixing blends.

SEM images of neat SEBS and SEBS/IL film blends evidence a surface morphology trend similar to that of PVC/IL films and are dependent on IL concentration (Figure 14). Neat SEBS film shows only small surface holes as peculiar features. These holes became larger in the SEBS blend membranes as the IL concentration increased, indicating that the IL loading contributes to the formation of surface defects. Moreover, the IL distribution is scattered on the film surfaces, showing aggregates of variable shape and size that tend to merge into large and compact structures. Nevertheless, these structures do not cover the film surfaces homogeneously even with the highest concentration of the additive. This could be due to the preparation of the SEBS blends. Indeed, during their preparation, we observed a partial loss of the IL additive in the mixing chamber. This loss was higher than that observed during the preparation of PVC/IL blends, indicating a lower compatibility between the SEBS matrix and the IL, as observed by DSC analysis. This lower compatibility is also in agreement with the decay of mechanical properties discussed above.

### 3.7. Contact Angle 

**Figure 15:**
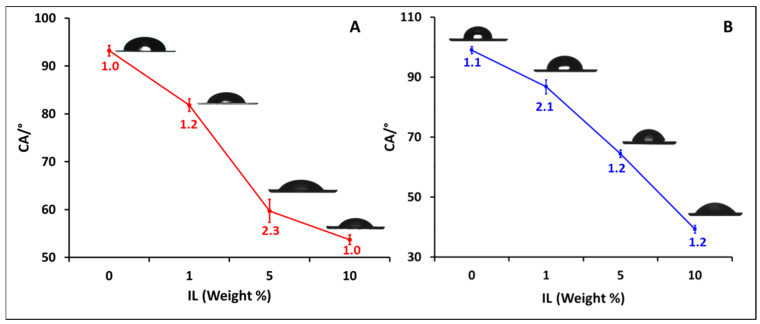
Water contact angle of (**A**) neat PVC and PVC/HdmimDMSIP blends, (**B**) neat SEBS and SEBS/HdmimDMSIP blends. Points are the mean of three independent replicates with n = 3 each. The points representing the CA values from PVC/1% IL and SEBS/1% IL blends had significantly different results from the related ones from both PVC and SEBS blends loaded with 5–10% of IL at *p* < 0.0001 (one-way ANOVA and Tukey test). The same result (*p* < 0.0001) was found when comparing the CA values from the IL blends with the related controls (neat polymers). Bars and numbers close to them represent ± SD.

The wettability of the membrane surfaces was determined by a static water contact angle. Measurements were performed at room temperature (ca. 25 °C) by dispensing water droplets onto three different locations of the specimen surface. The effect of the IL addition at different concentrations (1, 5, 10%) on the wettability of the polymer blends was analyzed. Both neat polymers are hydrophobic materials, displaying CA values for PVC and SEBS of 93.2° and 99.0°, respectively, whereas their IL blends showed a hydrophilic trend depending on IL concentration (Figure 15). 

The contact angle measurements can be mainly influenced by surface texture (roughness, particle shape, and size); indeed, the rise in surface roughness induces a CA increase or decrease according to the hydrophobicity/hydrophilicity of the neat materials [48]. Furthermore, IL structure and chemical composition can influence the blends’ wettability because the increase of the alkyl chain length increases cation hydrophobicity, whereas the H-bonding ability of the anions determines their hydrophilicity/hydrophobicity [49].

The PVC/IL and SEBS/IL blends were loaded with different concentrations of the same IL, thus their differences in CA principally depends on IL concentration and its miscibility/compatibility with the polymer matrix. The loading of the IL at high concentrations (5–10%) into both PVC and SEBS matrices induces surface roughness in their blends, as detected by SEM analysis. When the HdmimDMSIP concentration increased up to 10%, the water contact angle values of the PVC/IL films decreased from 93.2° to 53.7° (Figure 15A), while the loading of the same IL into the SEBS matrix induced a higher decrease of CA values from 99.0° to 39.3° (Figure 15B). Based on these results, it can be said that the IL concentration is decisive for surface morphology. The higher IL concentrations (5–10%) induce surface roughness. Nevertheless, the loading of the same IL concentration into different polymer matrices determines different values of contact angle, suggesting that polymer-IL compatibility is a key feature for wettability of the polymer blends containing the HdmimDMSIP IL. 

The HdmimDMSIP consists of a cation with a long alkyl chain, which makes it hydrophobic, and the DMSIP anion is less hydrophobic than other anions as PF_6_ [12]. The reduced CA values of its corresponding polymer blends confirm both cation and anion contribution to polymer wettability, as previously reported [12]. The specific orientation of the hydrophobic hexadecyl side chain toward the PVC matrix and the hydrophilic imidazolium ring and anion toward the polymer surface hypothesized in a previous work [12] is not well distinguished, perhaps due to the different preparation methods adopted. 

The neat SEBS membrane showed a high water contact angle of 99.0°. This value is close to those from 86° to 110° determined by Niu et al. [50] and Han et al. [51]. It was reported that an increase of the sulfonation degree induces a rapid decrease of the water contact angles of S-SEBS membranes. This is due to improved hydrophilicity of the S-SEBS membrane with the introduction of the hydrophilic sulfonic groups. S-SEBS membranes with a sulfonation degree of 54.1% showed a water contact angle of 23° [52].

### 3.8. IL Release

**Figure 16:**
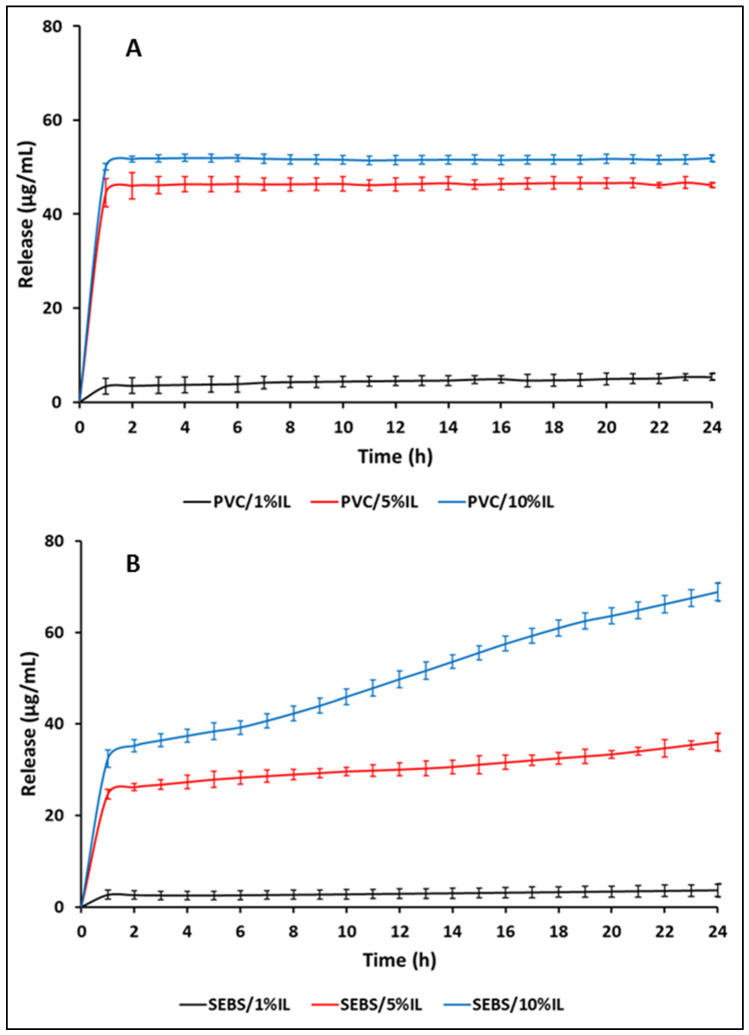
IL release from (**A**) PVC/HdmimDMSIP and (**B**) SEBS/HdmimDMSIP blends. Points are the mean of three independent replicates with n = 24 each. Bars represent ± SD.

The IL release in water from the blend films was investigated using UV-vis spectrophotometry. In Figure 16 and Figure S10, the release (µg/mL) from the PVC blends loaded with the IL at the concentrations of 1, 5, and 10% is reported. Results highlighted a fast IL release from the PVC/IL films, occurring within the first 2 h, with no further significant release up to 24 h (Figure 16A). Comparing the blends containing the IL at 1% and 10% concentrations, a dose-dependent release was found. In particular, the release from PVC/1% IL showed increasing values of 3.1, 4.2, and 5.4 µg/mL after 1, 7 and 24 h, respectively, with a mean value of 4.4 ± 0.6 µg/mL. In the case of both PVC/5% IL and PVC/10% IL membranes, a massive release was found during the first 3 h of analysis and only slight variations in the following 21 h. This trend suggests that during the first 3 h a saturation of IL in the water solution occurred, reaching an equilibrium state in the following period of observation. It is confirmed by the release values of 44.6 and 50.7 after 1 h and 46.4 and 51.9 recorded at 24 h from PVC/5% IL and PVC/10% IL, respectively.

The IL release from the SEBS blends was dose- and time-dependent (Figure 16B), showing values of 3.8, 36.2, and 68.8 µg/mL at 24 h as IL concentration increases in the blends. The release from the SEBS blends containing the 5% of IL was half of that containing the 10% of IL, thus confirming the dose-dependent release. On the contrary, the release from PVC/IL blends at 5–10% IL concentration is very similar, suggesting that these amounts are excessive for compatibilization with the PVC matrix, as previously reported (12). This is confirmed by SEM analysis (Figure 13). The IL release from the SEBS blends seems to be favored by the peculiar holes observed on their surfaces (Figure 14) that allow a progressive IL release over time. This feature could be interesting for the development of SEBS-based drug delivery materials. Statistical analysis (One-way ANOVA and Tukey’s multiple comparisons test) highlighted a significant difference at *p* < 0.0001 between each PVC/IL blend vs. each other and vs. the control (neat PVC), except between control vs. PVC/1% IL and PVC/5% IL vs. PVC/10% IL, both showing a significant difference at *p* < 0.01. The comparison between neat SEBS vs. SEBS/1% IL displayed a significant difference at *p* < 0.01, whereas both SEBS/5% IL and SEBS/10% IL blends resulted significantly different from the control and SEBS/1% IL at *p* < 0.0001. Moreover, a significant difference at *p* < 0.001 was found between neat SEBS vs. SEBS/5% IL and SEBS/5% IL vs. SEBS/10% IL.

### 3.9. Antibacterial Activity

Bacterial infections and antibiotic-resistant bacteria are key issues that cause serious problems for public health and safety. They have stimulated the search for biocide alternative to antibiotics, as many diseases are rising due to the contamination of products used in everyday life, causing mortality and morbidity throughout the world. Moreover, the demand for the development of antimicrobial materials to be used for different applications is on the rise, including food packaging and biomedical fields. Nevertheless, for a fast and easy loading of antimicrobial agents into polymeric matrices, it is necessary that the biocides used in industrial manufacturing processes are thermally stable. For this reason, both HdmimBr and HdmimDMSIP ILs were analyzed to determine their thermal stability. As previously reported, the hygroscopic behavior of HdmimBr affects its thermal stability, hindering its loading into PVC and SEBS matrices due to its low T_onset_, very close to temperatures used during melt-mixing. As a consequence, only the antibacterial activity of PVC and SEBS blends containing HdmimDMSIP was analyzed. 

The antibacterial activity of the neat HdmimDMSIP was determined using the broth dilution method. The IL showed the same value (10 µg/mL) of minimum inhibitory concentration (MIC) and minimum bactericidal concentration (MBC) against *S. epidermidis*, while the MIC and MBC values vs. *E. coli* were of 100 and 500 µg/mL, respectively.

The potential antibacterial activity of the neat polymers and their IL blend films against *S. epidermidis* and *E. coli* growth was determined using the agar diffusion method. Round samples (diameter 2.15 cm, thickness 120–150 µm) were used. The PVC/IL and SEBS/IL blend films showed inhibition of *S. epidermidis* growth at all IL concentrations (1, 5, and 10%), whereas their activity vs *E. coli* growth highlighted inhibition only at high concentrations (5% and 10%) of IL (Table 2). 

The values of inhibition zones found for both PVC/IL and SEBS/IL (Figure S11) blends are in good agreement with data from SEM, contact angle, and release analyses. Indeed, the PVC blends containing 5% and 10% of IL showed values very similar due to the accumulation of IL on the film surfaces (Figure 13) and its consequent fast release within the first 3 h (Figure 16A). Conversely, the SEBS IL blends displayed dose-dependent values, in accordance with the SEM, contact angle, and release data (Figure 14, Figure 15 and Figure 16B).

In a previous study regarding the preparation of imidazolium PBT ionomers with an ionic group located randomly along the polymer chain or selectively as an end-group (telecheric), Colonna and co-authors [26], testing the same IL, found that the synthetized imidazolium ionomers showed antimicrobial activity against *S. aureus* ATCC 6538 and *E. coli* ATCC 25645 strains comparable with that of commercial antimicrobial agents such as Triclosan. 

Considering the antimicrobial activity of the neat HdmimDMSIP, the results showed excellent activity vs. *S. epidermidis*, and lower activity vs. *E. coli*, suggesting a lower sensitivity in the latter strain. The IL loading at different concentrations into different polymer matrices [11,12,26] highlighted different behaviors depending on the structure and the physical-chemical properties of the tested polymers, which may facilitate the release of IL and consequently its diffusion in TSA. 

In vitro analyses of the properties of 1-alkyl-3-methylimidazolium chloride ionic liquids showed that increasing the alkyl chain length of ILs increased the anti-biofilm activity [53,54]. This phenomenon is clearly seen in Gram-positive coccaceae, which were inhibited in at least three concentrations of N-cinnamylalkylimidazolium ILs. Likewise, the same phenomenon was observed in *E. coli*, to a lesser extent [54]. The inhibition halos in the presence of *E. coli*, indeed, were usually smaller than those observed for *S. epidermidis*, confirming a higher sensitivity of Gram-positive bacteria to the antimicrobial activity of HdmimDMSIP. These results are in agreement with previous studies reporting a higher toxicity of imidazolium blends against Gram-positive bacteria and a tolerance against Gram-negative strains [17,18,49].

It was hypothesized that Gram-negative bacteria can express multiple resistance factors towards ILs and also towards the antibiotics used for the treatment of infections [55]. It is also reported that Gram-negative bacteria can express molecular expulsion pumps and modify their target sites to block drugs; in addition, they can also express hydrolytic enzymes and membrane proteins that would show resistance to osmotic shock [56,57]. 

Considering their antimicrobial activity, the developed PVC/IL and SEBS/IL blend films, reducing bacterial contamination, could find applications in different fields.

## 4. Conclusions

The aim of this study was the development of antibacterial polymer blends based on PVC and SEBS, loaded with the ionic liquid HdmimDMSIP at different concentrations (1, 5, and 10%). The IL/blends were characterized by their thermo-mechanical properties, surface morphology, and wettability. IL release from the blends and their antibacterial activity against *S. epidermidis* and *E. coli* were evaluated. The results suggested compatibility between the IL and the PVC matrix, whereas phase separation was detected in the SEBS/IL blends. TGA and PY-GC MS analyses highlighted that the loading of the IL into the PVC matrix influences the dehydrochlorination process occurring during the first thermal degradation step of the polymer. The IL addition in this polymer also determines the lowering of the thermal stability of the TOTM plasticizer present in the PVC matrix. The mechanical analysis evidenced a dose-dependent lowering of Young’s Modulus, Tensile Stress, and Strain at Break in the SEBS/IL blends. The PVC/IL blends showed a similar trend, but with an increase of the Strain at Break as the IL concentration in the blends increased, suggesting a plasticizer effect. SEM analyses underlined a different IL surface distribution in the blends, with abundant IL deposition on PVC blends and irregular structures similar to islands in the SEBS/IL films. The water contact angle analysis demonstrated that the loading of the IL into both polymer matrices induces higher wettability of their blend surfaces, in particular in the SEBS films. The determination of the IL release from the blends highlighted a dose-dependent behavior and also a time-dependent one from the SEBS/IL blends. Both PVC/IL and SEBS/IL blends exhibited the best antimicrobial activity against *S. epidermidis*, being active at low concentration (1%), whereas the antimicrobial activity against *E. coli* was minor. 

These results indicate that these new materials are promising for a versatile use in a variety of fields.

## Figures and Tables

**Table 1:** Mechanical properties (Young’s Modulus E; Tensile Stress σ; Strain at Break [%] ε) of the PVC/IL and SEBS/IL blends are reported as mean values of PVC/IL (n = 5) and SEBS/IL (n = 6) replicates ± SD. A statistical comparison between the different groups and each of them with their related controls (neat polymers) was performed by One-Way ANOVA with Tukey’s multiple comparisons test. *** *p* < 0.001; **** *p* < 0.0001.

Samples	E, Young’s Modulus [Mpa]	σ, Tensile Stress [Mpa]	ε, Strain at Break [%]
PVC	12.00 ± 0.781	13.68 ± 0.33	264.70 ± 14.7
PVC/1% IL	9.81 ± 0.667 ***	13.00 ± 0.39	289.60 ± 16.6
PVC/5% IL	7.39 ± 0.199 ****	10.36 ± 1.58 ***	294.90 ± 38.8
PVC/10% IL	7.19 ± 0.361 ****	10.16 ± 1.05 ***	317.70 ± 44.2
SEBS	24.60 ± 1.270	20.73 ± 0.39	1287.90 ± 26
SEBS/1% IL	23.20 ± 2.150	20.04 ± 0.40	1139.60 ± 49 ***
SEBS/5% IL	22.40 ± 2.000 ***	19.84 ± 0.67	1120.00 ± 22 ***
SEBS/10% IL	21.20 ± 1.320 ***	18.26 ± 1.00 ****	1111.10 ± 30 ***

**Table 2:** Inhibition zones induced by PVC and SEBS films loaded with different percentages of 1-hexadecyl-3-methyl imidazolium 1.3-dimethyl 5-sulfoisophthalate (HdmimDMSIP). The values are mean of three independent replicates. Bars represent ± SD. A statistical comparison between the different groups and between each of them and their related controls (neat polymers) was performed by One-Way ANOVA with Tukey’s multiple comparisons test. *** *p* < 0.001; **** *p* < 0.0001.

Sample	*S. epidermidis* Halos (mm) ± SD ^a^	*E. coli* Halos (mm) ± SD ^a^
Neat PVC	NI	NI
PVC/1% HdmimDMSIP	3.7 ± 1.2 ***	NI
PVC/5% HdmimDMSIP	5.3 ± 2.1 ****	2.0 ± 1.4
PVC/10% HdmimDMSIP	5.7 ± 1.2 ****	2.0 ± 1.0
Neat SEBS	NI	NI
SEBS/1% HdmimDMSIP	2.0 ± 0.1 ***	NI
SEBS/5% HdmimDMSIP	4.5 ± 0.7 ****	2.0 ± 0.1 ***
SEBS/10% HdmimDMSIP	8.0 ± 0.1 ****	3.0 ± 0.1 ***

^a^ Mean of three independent replicates ± standard deviation. NI = no inhibition.

## Supplementary Materials

The following supporting information can be downloaded at: https://www.mdpi.com/article/10.3390/polym15040970/s1, Table S1. Structural assignments of cations and adducts identified in the MALDI-TOF mass spectra of the ILs 1-hexadecyl-3-methylimidazolium bromide (HdmimBr) and 1-hexadecyl-3-methylimidazolium 1,3-dimethyl 5-sulfoisophthalate (HdmimDMSIP); Figure S1. TGA of the IL 1-hexadecyl-3-methylimidazolium bromide (HdmimBr); Figure S2. TGA of the IL 1-hexadecyl-3-methylimidazolium 1,3-dimethyl 5-sulfoisophthalate (HdmimDMSIP); Figure S3. DSC of the IL 1-hexadecyl-3-methylimidazolium bromide (HdmimBr). Curves are displaced for clarity; Figure S4. DSC curves of the IL 1-hexadecyl-3-methylimidazolium 1,3-dimethyl 5-sulfoisophthalate (HdmimDMSIP). Curves are displaced for clarity; Figure S5. Py-GCMS pyrograms of the pyrolysis products of the PVC/1% HdmimDMSIP blend at (a) 300 °C and (b) 450 °C; Figure S6. Py-GCMS pyrograms of the pyrolysis products of the PVC/5% HdmimDMSIP blend at (a) 260 °C, (b) 300 °C and (c) 350 and (d) 450 °C; Figure S7. Py-GCMS pyrograms of the pyrolysis products of the neat SEBS (a) and SEBS/10% HdmimDMSIP blend (b) both at 300 °C. The inset shows the fragmentation products from the HdmimDMSIP; Figure S8. Py-GCMS pyrograms of the pyrolysis products of the SEBS/5% HdmimDMSIP blend at (a) 300 °C and (b) 450 °C; Figure S9. Mechanical properties (Young’s modulus, Tensile strength and Elongation at break percentage) of the PVC/HdmimDMSIP (a,b,c) and SEBS/HdmimDMSIP (d–f) blends as HdmimDMSIP % increases; Figure S10. HdmimDMSIP release from A) PVC/HdmimDMSIP and B) SEBS/HdmimDMSIP blends. Each point represents the HdmimDMSIP release from the PVC/HdmimDMSIP and SEBS/HdmimDMSIP blends at fixed times (1-24 h); Figure S11. Inhibion haloes induced by the SEBS/HdmimDMSIP blends, loaded with three different concentration of HdmimDMSIP (0.5%, 1%, 5%), on TSA seeded with S. epidermidis (106 CFU/ml).
