# Supplementary material for: Polymer Blends Based on 1-Hexadecyl-3-methyl Imidazolium 1,3-Dimethyl 5-Sulfoisophthalate Ionic Liquid: Thermo-Mechanical, Surface Morphology and Antibacterial Properties
Source: Polymers (Basel). 2023 Feb 16;15(4):970. doi: 10.3390/polym15040970 (PMC9965557; doi:10.3390/polym15040970)
Supplement: Supplementary file 1 [file polymers-15-00970-s001.zip › polymers-2166885-supplementary.pdf]

Supplementary

Polymer Blends Based on 1-Hexadecyl-3-Methyl Imidazolium 1,3-Dimethyl 5-Sulfoisophthalate Ionic Liquid: Thermo-Mechanical, Surface Morphology and Antibacterial Properties

Daniela C. Zampino [1,*], Filippo Samperi [1,*], Monique Mancuso [2,3], Tiziana Ferreri [4], Loredana Ferreri [4], Sandro Dattilo [1], Emanuele F. Mirabella [1], Domenico C. Carbone [1], Giuseppe Recca [1], Andrea A. Scamporrino [1], Elisabetta Novello [1] and Concetto Puglisi [1]

[1] Institute of Polymers, Composites and Biomaterials (IPCB)-CNR, Section of Catania, Via Paolo Gaifami, 18, 95126 Catania, Italy
[2] Institute for Biological Resources and Marine Biotechnology (IRBIM)-CNR, Section of Messina, Spianata San Raineri, 86, 98122 Messina, Italy
[3] Department of Integrative Marine Ecology, Stazione Zoologica "Anton Dohrn", Sicily Marine Centre, Contrada Porticatello, 29, 98167 Messina, Italy
[4] Institute of Biomolecular Chemistry (ICB)-CNR, Section of Catania, Via Paolo Gaifami, 18, 95126 Catania, Italy
* Correspondence: danielaclotilde.zampino@cnr.it (D.C.Z.); filippo.samperi@cnr.it (F.S.)

Table S1. Structural assignments of cations and adducts identified in the MALDI-TOF mass spectra of the ILs 1-hexadecyl-3-methylimidazolium bromide (HdmimBr) and 1-hexadecyl-3-methylimidazolium 1,3-dimethyl 5-sulfoisophthalate (HdmimDMSIP).

Symbols	Structures	Measured m/z	Calculated m/z
**Hdmim$^+$**	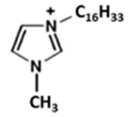	**307.4071**	**307.3113**
**Hdmim$^+$Br$^-$ + Hdmim$^+$**	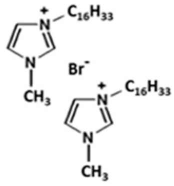	**694.994**	**694.978**
**Hdmim$^+$**	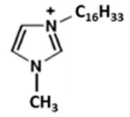	**307.3396**	**307.3113**
**Hdmim$^+$DMSIP$^-$ + HDMIm$^+$**	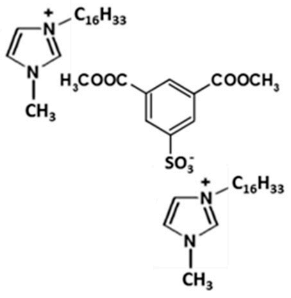	**887.7729**	**887.6296**

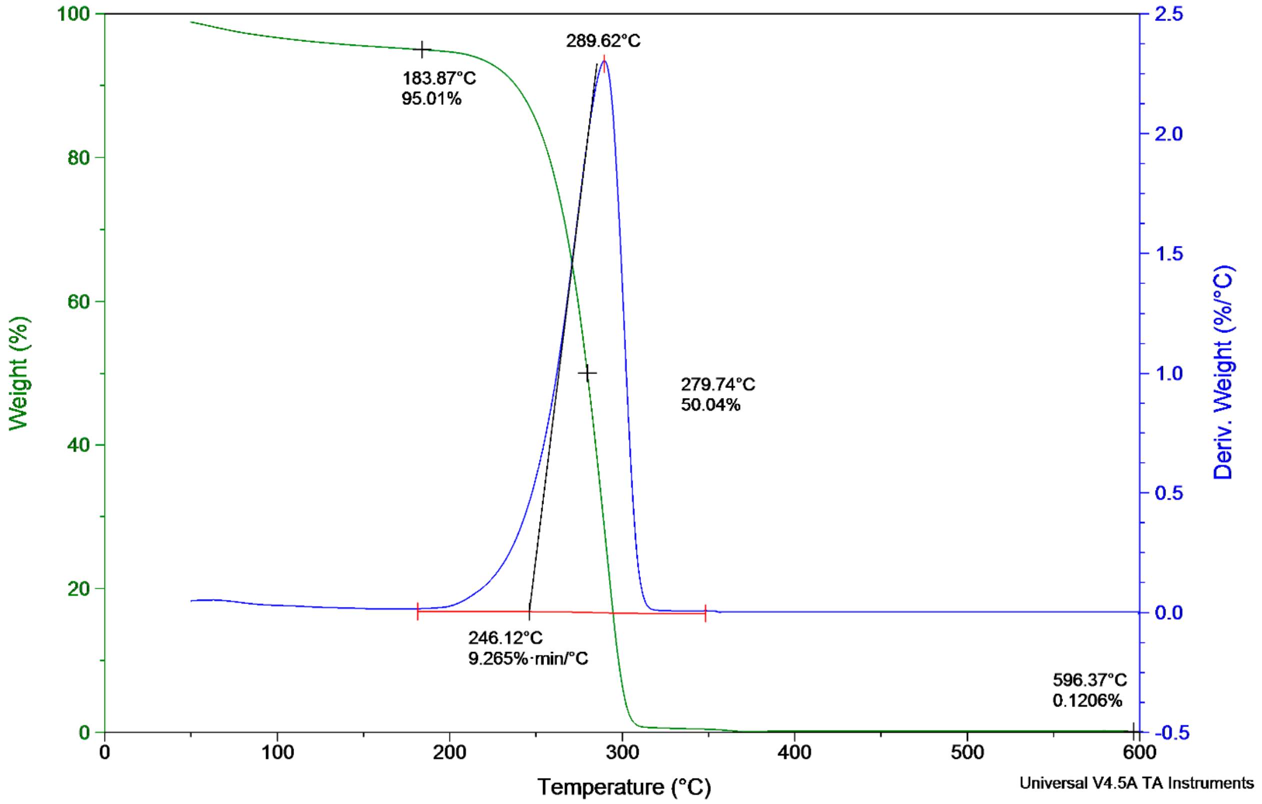

Figure S1. TGA of the IL 1-hexadecyl-3-methylimidazolium bromide (HdmimBr).

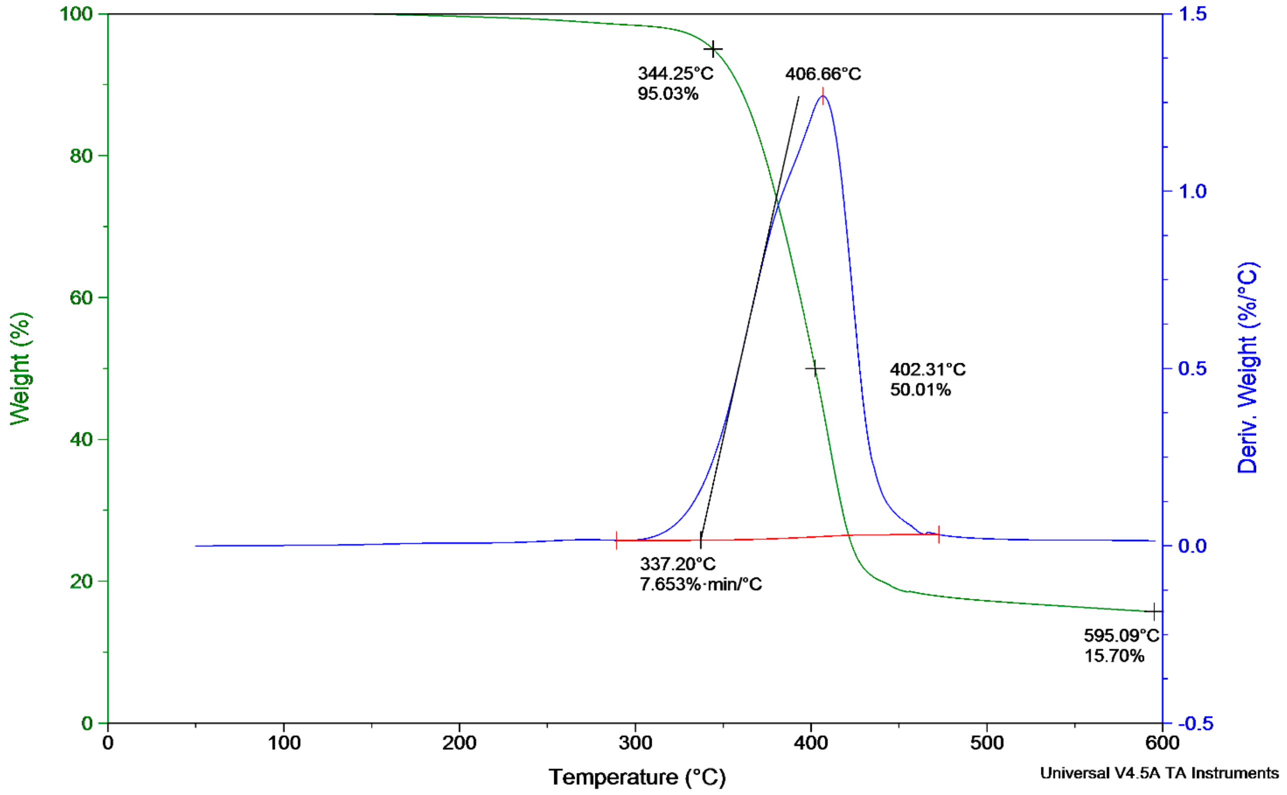

Figure S2. TGA of the IL 1-hexadecyl-3-methylimidazolium 1,3-dimethyl 5-sulfoisophthalate (HdmimDMSIP).

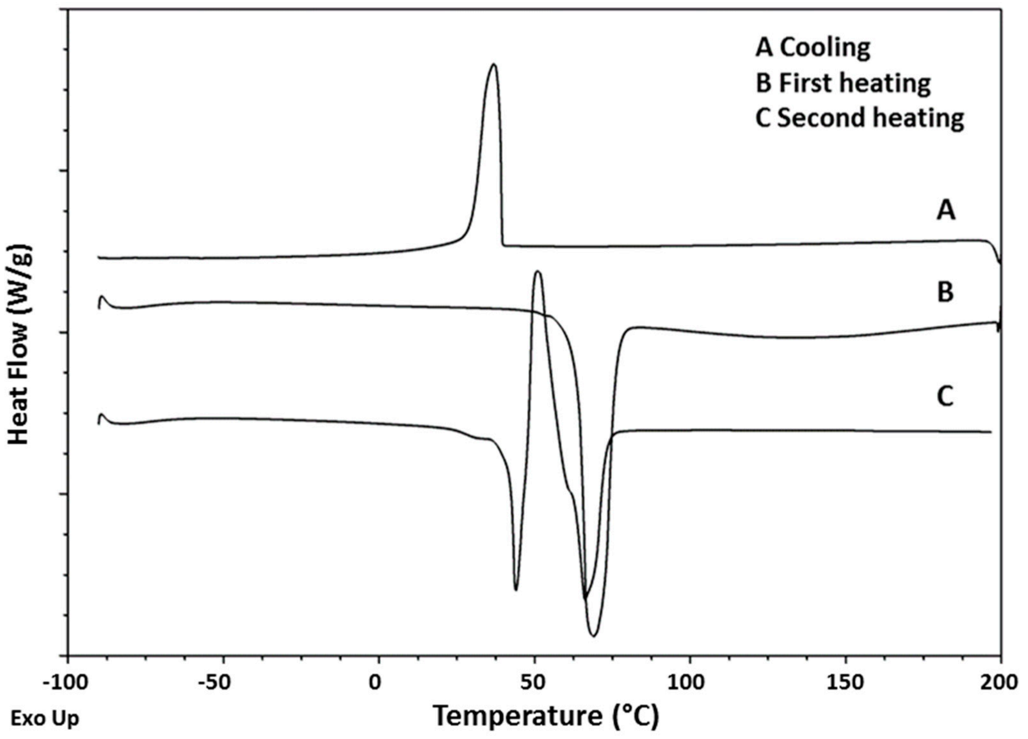

Figure S3. DSC of the IL 1-hexadecyl-3-methylimidazolium bromide (HdmimBr). Curves are displaced for clarity.

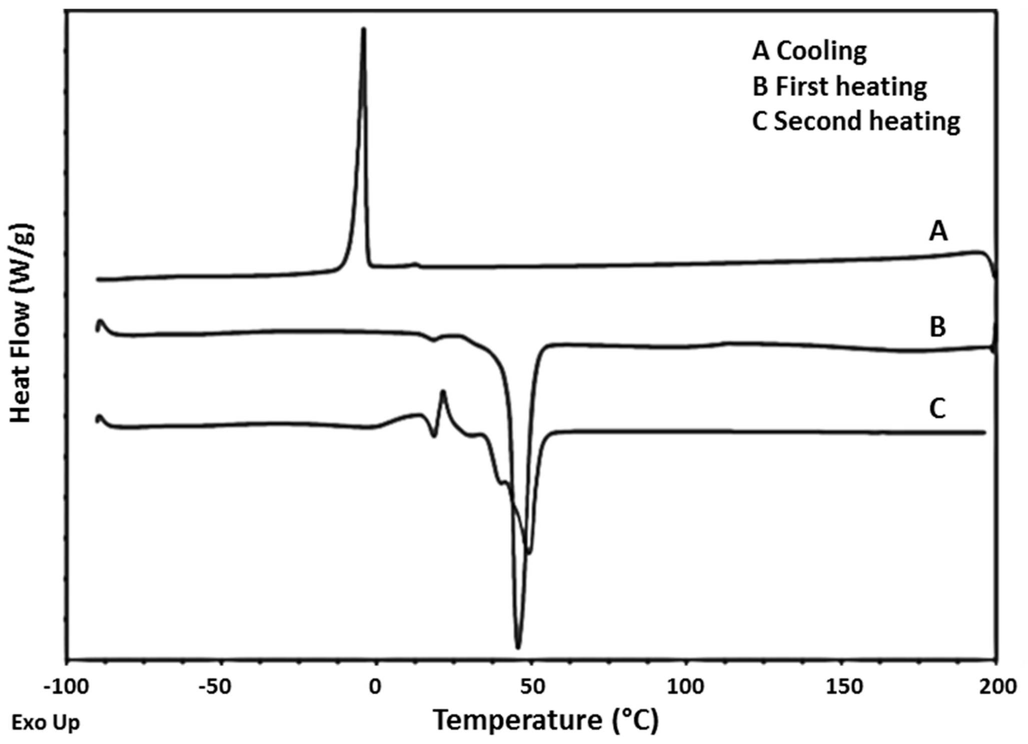

Figure S4. DSC curves of the IL 1-hexadecyl-3-methylimidazolium 1,3-dimethyl 5-sulfoisophthalate (HdmimDMSIP). Curves are displaced for clarity.

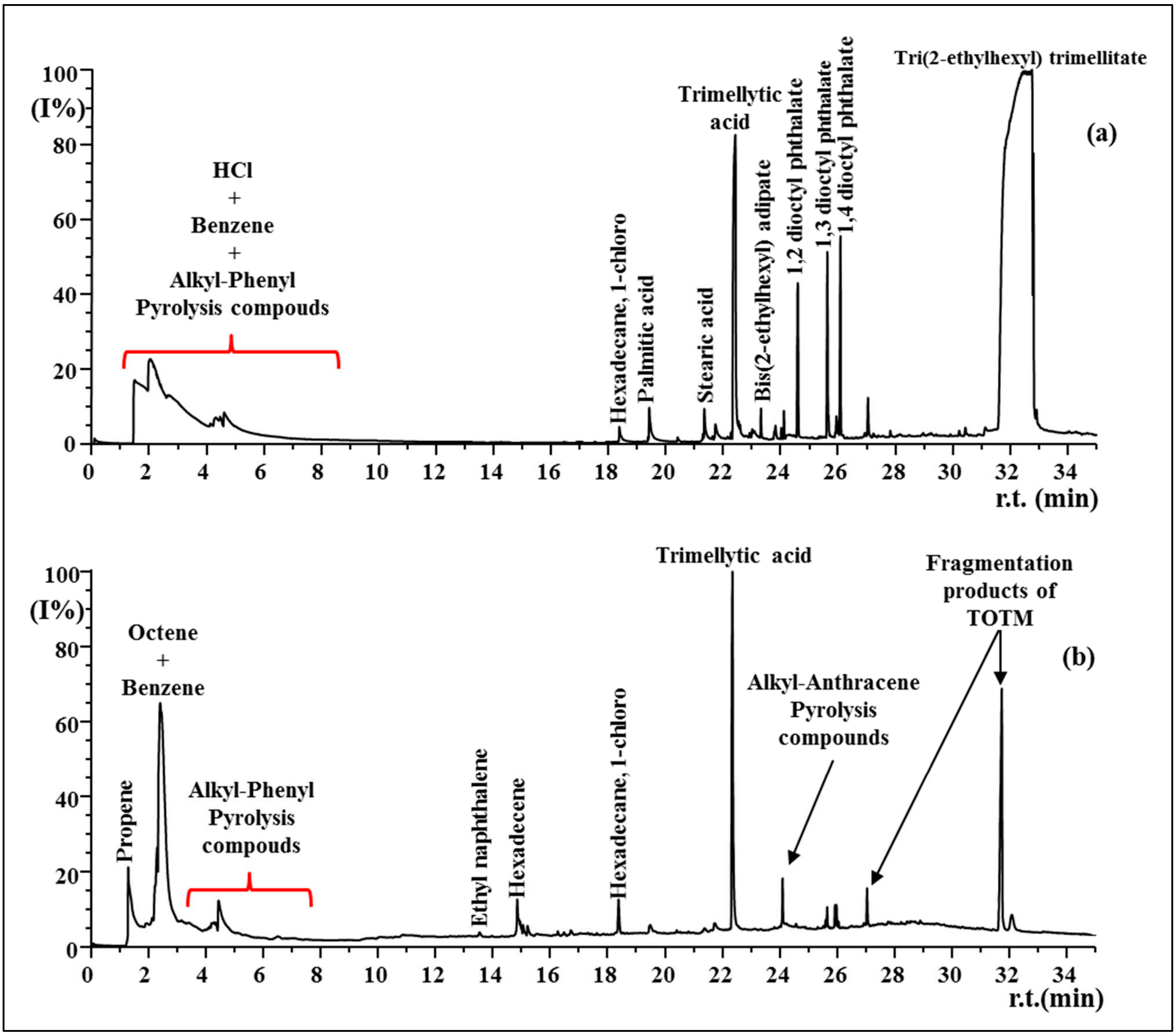

Figure S5. Py-GCMS pyrograms of the pyrolysis products of the PVC/1% HdmimDMSIP blend at (**a**) 300°C and (**b**) 450 °C.

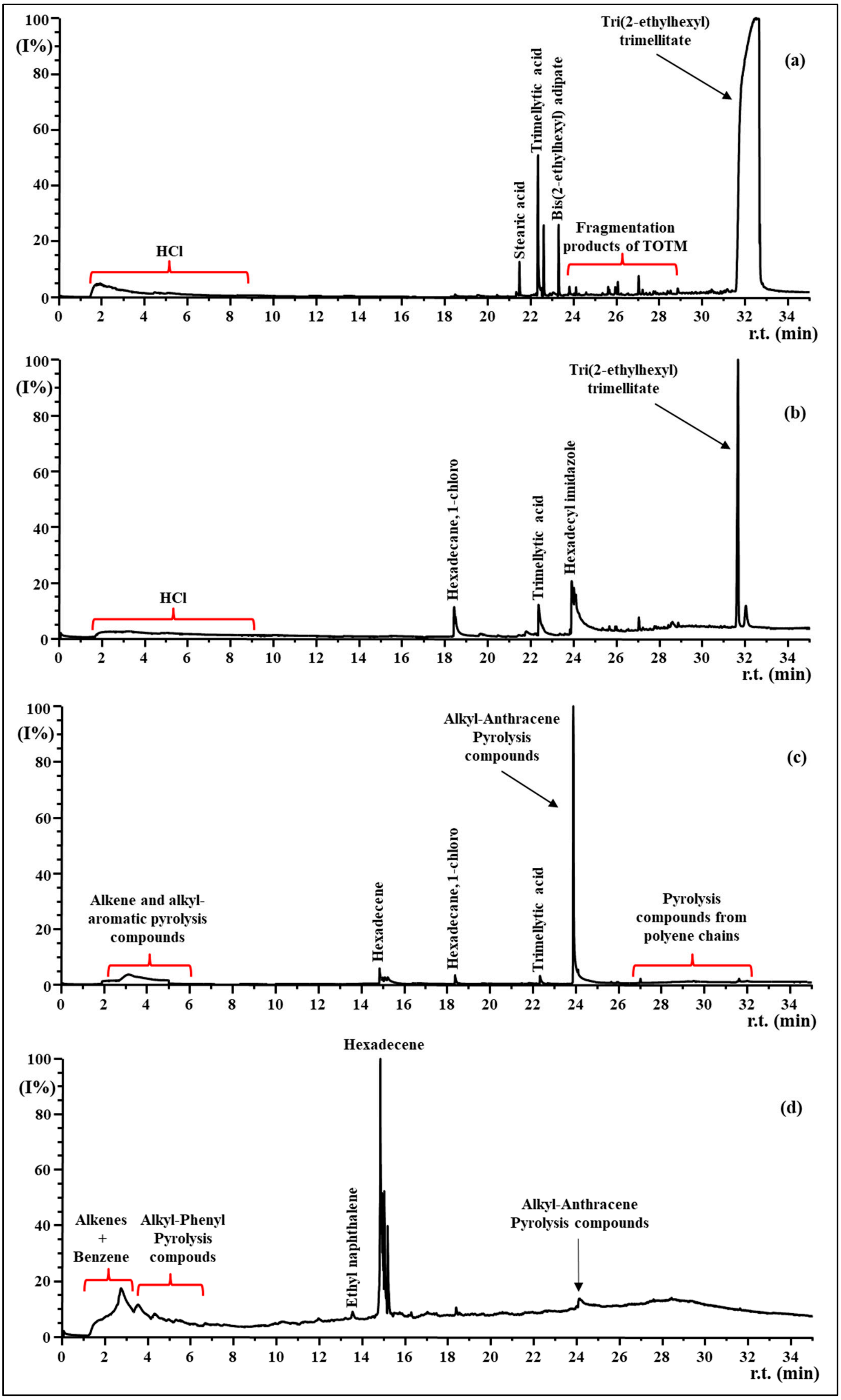

Figure S6. Py-GCMS pyrograms of the pyrolysis products of the PVC/5% HdmimDMSIP blend at (**a**) 260°C, (**b**) 300°C and (**c**) 350 and (**d**) 450 °C.

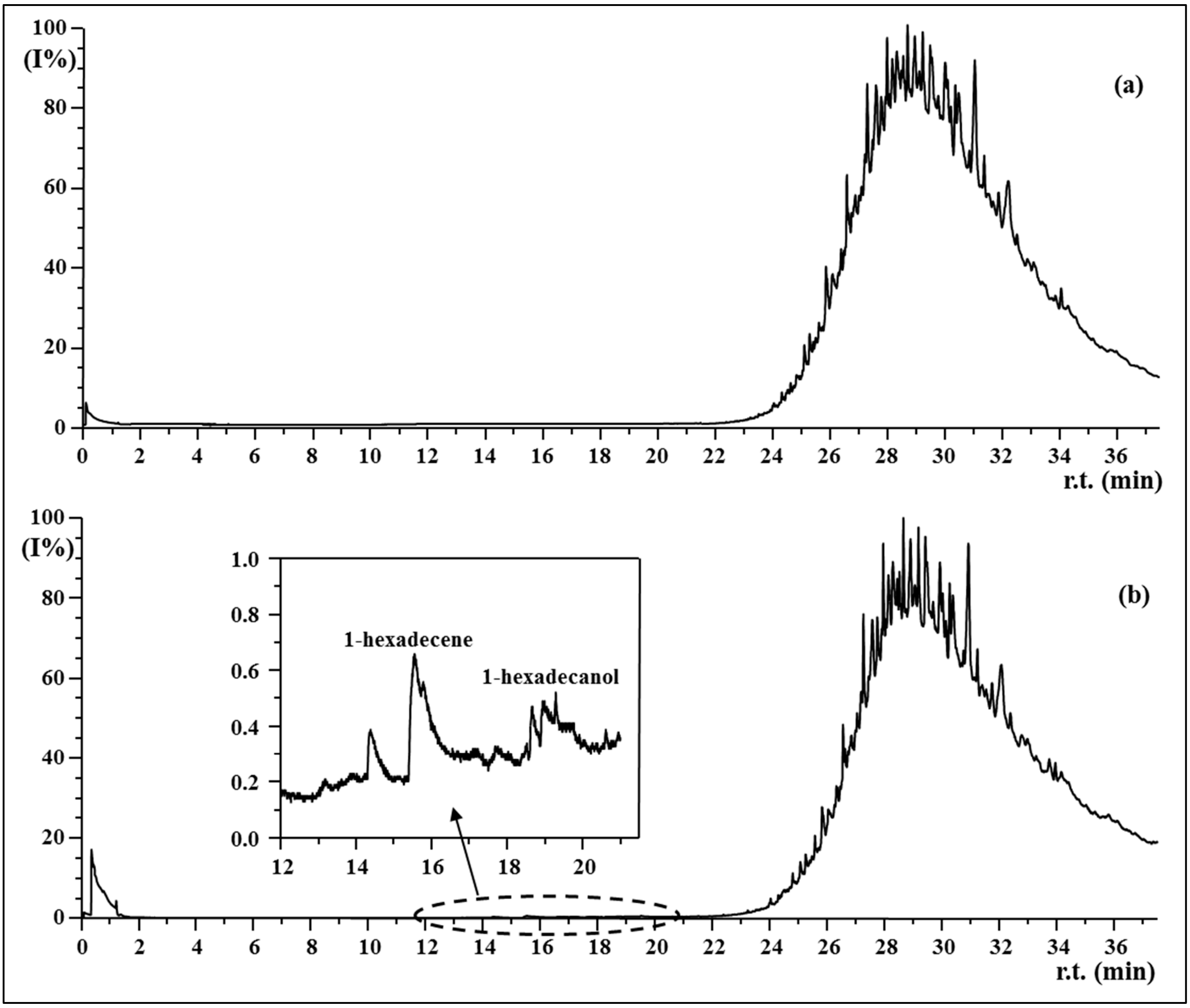

Figure S7. Py-GCMS pyrograms of the pyrolysis products of the neat SEBS (**a**) and SEBS/10% HdmimDMSIP blend (**b**) both at 300°C. The inset shows the fragmentation products from the HdmimDMSIP.

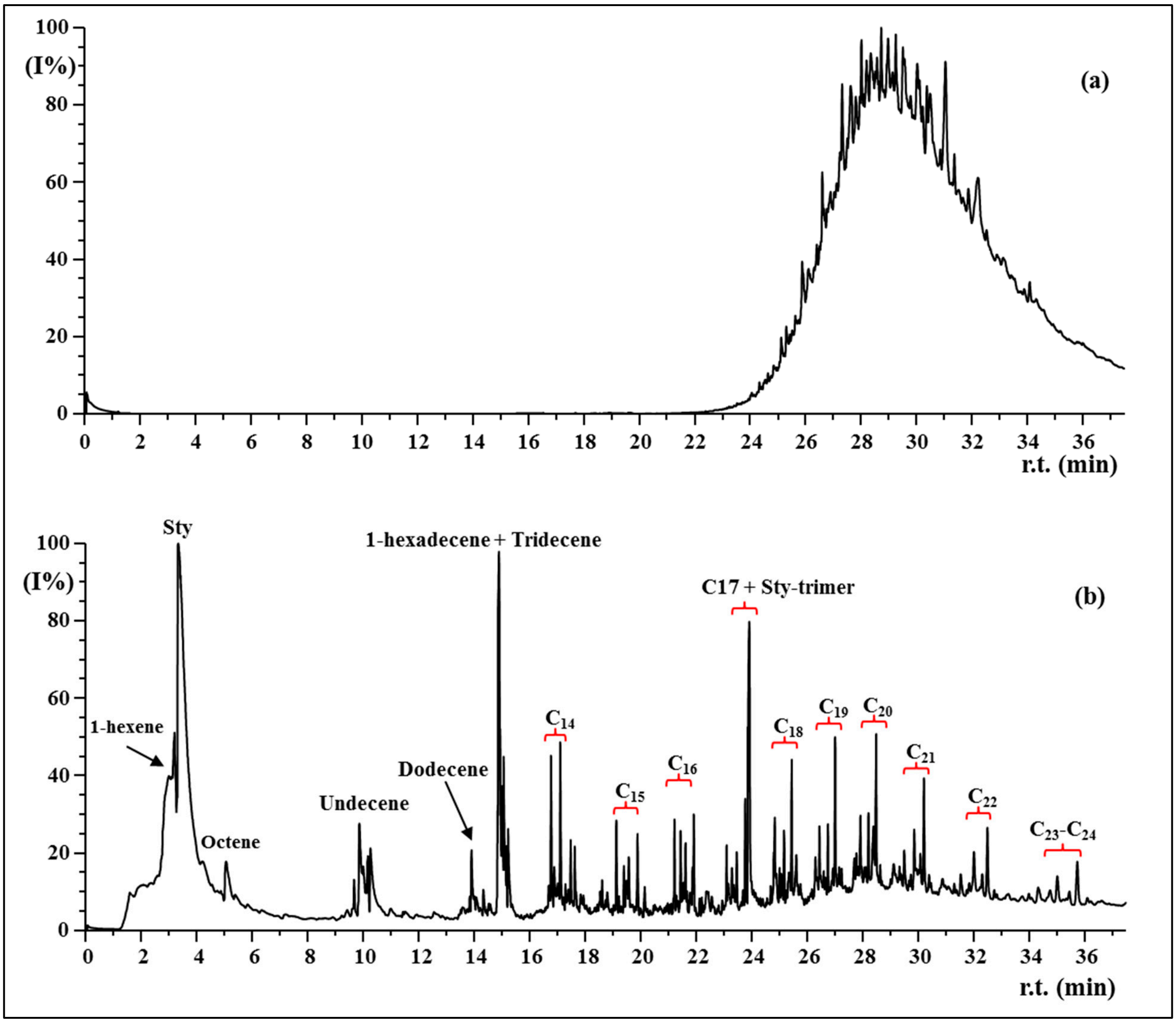

Figure S8. Py-GCMS pyrograms of the pyrolysis products of the SEBS/5% HdmimDMSIP blend at (**a**) 300°C and (**b**) 450 °C.

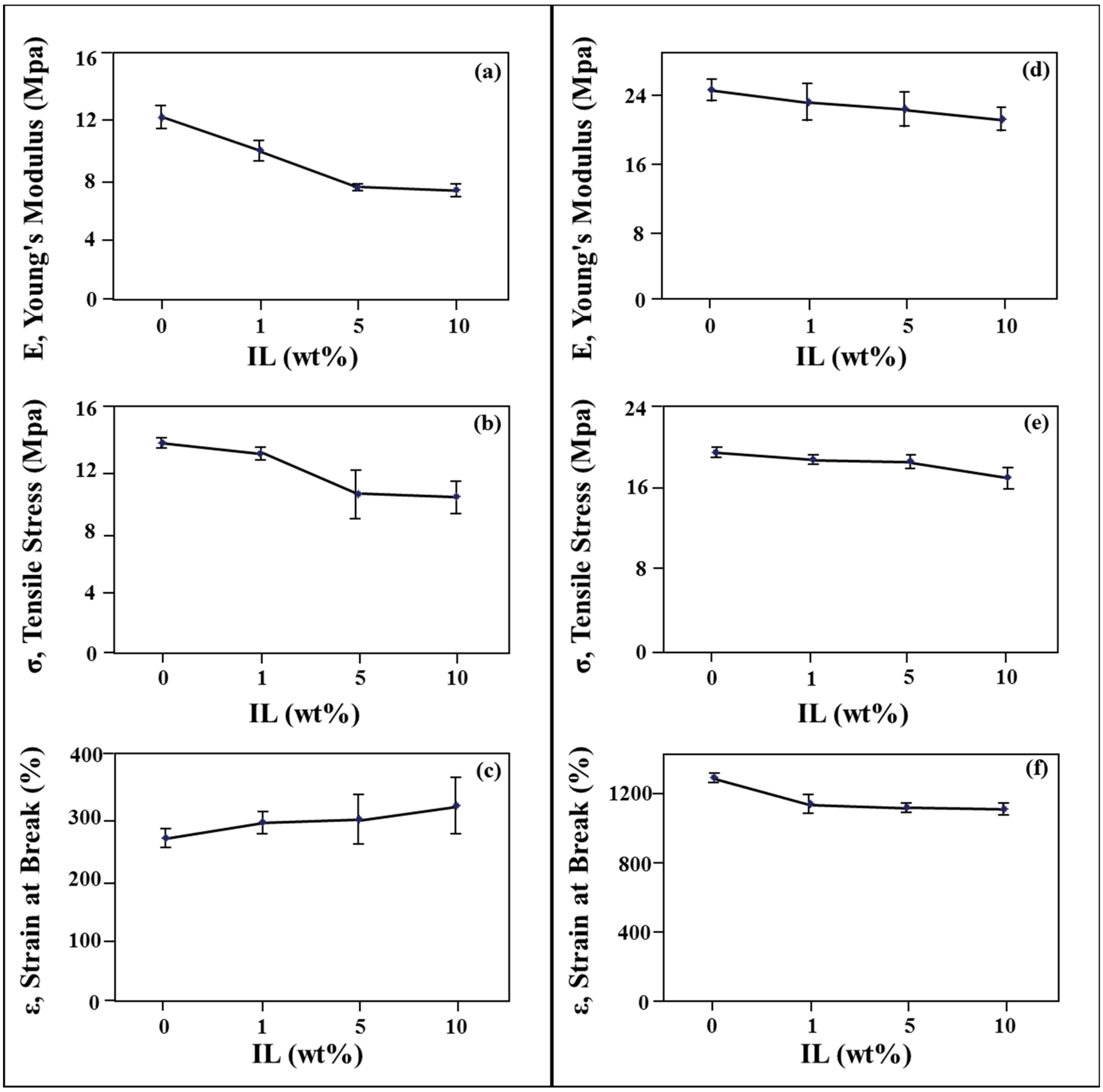

Figure S9. Mechanical properties (Young's modulus, Tensile strength and Elongation at break percentage) of the PVC/HdmimDMSIP (**a**,**b**,**c**) and SEBS/HdmimDMSIP (**d**, **e**, **f**) blends as HdmimDMSIP % increases.

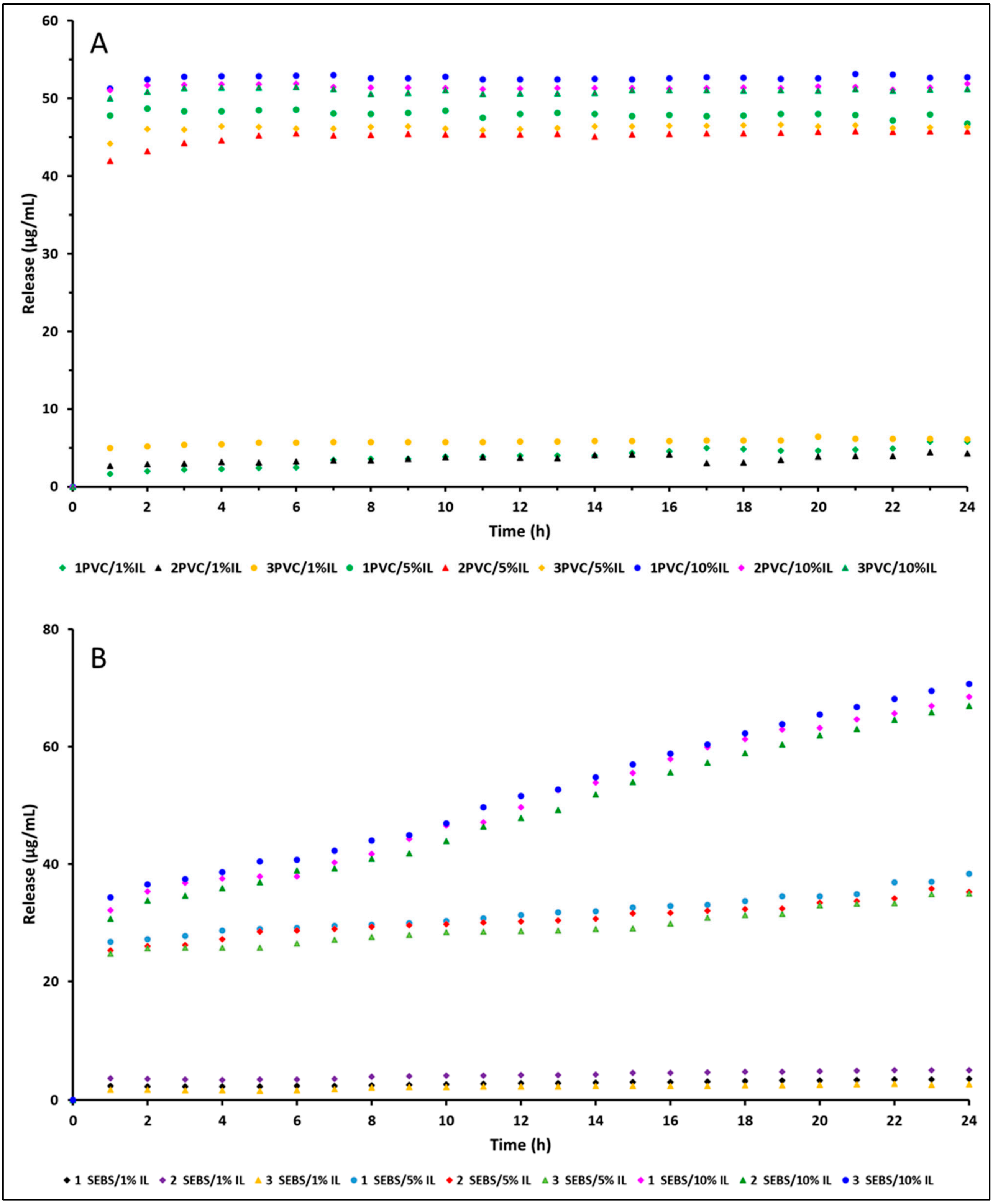

Figure S10. IL release from (**A**) PVC/HdmimDMSIP and (**B**) SEBS/HdmimDMSIP blends. Each point represents the HdmimDMSIP release from the PVC/HdmimDMSIP and SEBS/HdmimDMSIP blends at fixed times (1-24 h).

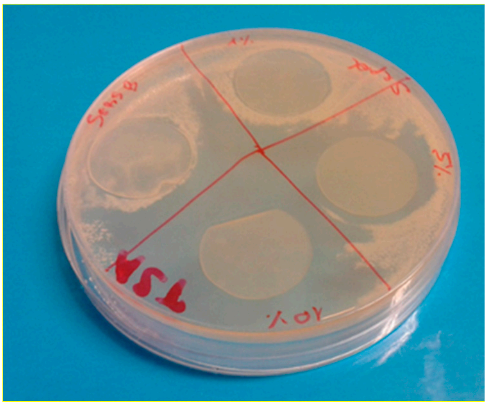

Figure S11. Inhibion haloes induced by the SEBS/HdmimDMSIP blends, loaded with three different concentration of HdmimDMSIP (0.5%, 1%, 5%), on TSA seeded with *S. epidermidis* (10^6 CFU/ml).
